# The locomotor ecology of wild western lowland gorillas: How does the largest ape exploit complex arboreal environments?

**DOI:** 10.1111/joa.14277

**Published:** 2025-05-09

**Authors:** Charlotte A. King, Jackie Chappell, Martha M. Robbins, Robin H. Crompton, William I. Sellers, Susannah K. S. Thorpe

**Affiliations:** ^1^ School of Biosciences University of Birmingham Birmingham UK; ^2^ Department of Primate Behavior and Evolution Max Planck Institute for Evolutionary Anthropology Leipzig Germany; ^3^ Department of Musculoskeletal and Ageing Science, Institute of Life Course & Medical Sciences University of Liverpool Liverpool UK; ^4^ Department of Earth and Environmental Sciences University of Manchester Manchester UK

**Keywords:** arboreality, body size, energy, evolution, *Gorilla gorilla gorilla*, locomotion

## Abstract

Western lowland gorillas are the largest and most sexually dimorphic ape that habitually exploits arboreal environments. Their size, robust musculature and specialised adaptations in the hands and feet, which are suited for terrestrial quadrupedal locomotion, make them interesting models for understanding how great apes are able to exploit complex arboreal habitats. We present a comprehensive analysis of the arboreal locomotor ecology of western lowland gorillas by studying their behaviour and ecology in the context of their morphology. A group of fully habituated wild western lowland gorillas was followed for 12 months in Loango National Park, Gabon. Statistical analysis applying regression modelling and Akaike's Information Criterion was used to identify the relationships between locomotor behaviours, height, contextual behaviour, support use, hand posture and body size. Our findings suggest that the gorillas were not restricted in their ability to access and move around in tree canopies because of their size or postcranial morphology. Instead, they exhibited considerable behavioural flexibility and engaged in locomotor behaviours that contradicted classic body size predictions for primates. To offset the risks of moving on small supports, the gorillas used hand‐assisted bipedal locomotion on multiple small supports, rather than relying on suspensory locomotion. We suggest that this is linked to their hand dimensions, which have been selected to facilitate efficient quadrupedal walking on the ground. The silverback gorilla engaged in less horizontal locomotion in the canopy, spent less time at heights above 20 m, and used large supports more often than the adult females, blackback and adolescents, but the type and number of supports used did not vary between body size groups. We also found that the reproductive status of the females (presence or absence of small infants) may have shaped how they responded to risks when solving the problem of gap‐crossing in the trees. Overall, our results highlight that the gorillas likely prioritised risk minimisation in the supports that they used in arboreal environments at the cost of increased energy expenditure.

## Graphical abstract

When travelling and feeding in the canopy, the western lowland gorillas engaged in bipedal locomotion likely as a strategy to mitigate the risk of falling at the cost of increased energy expenditure.

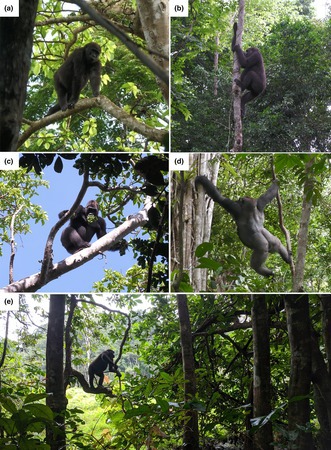

## 1 INTRODUCTION

Western lowland gorillas (*Gorilla gorilla gorilla*) are the largest and most sexually dimorphic ape that habitually exploits arboreal environments. Silverback gorillas weigh up to 190 kg, which is more than twice the weight of adult females, which weigh on average 70 kg (Jungers & Susman, 1984; Wilhoughby, 1978; Zihlman & McFarland, 2000). This is more diverse than the almost exclusively arboreal orangutans (with males weighing 90 kg and females weighing 45 kg) (Knott, 1998; Rodman, 1984). However, while the positional behaviour (locomotion and posture) of orangutans has been well studied (Cant, 1987; Manduell et al., 2012; Thorpe et al., 2009; Thorpe & Crompton, 2005), there is very little data for gorillas in the wild. In particular, it is not fully understood how, with adaptations towards terrestrial quadrupedal walking and such large body size, western lowland gorillas move in the trees to feed on arboreal resources.

The western lowland gorillas in Loango National Park, Gabon, inhabit a tropical rainforest which is characterised by an array of differing habitat types, including coastal forests, savannahs, mangroves, swamps and primary forest (Harris et al., 2012; Head et al., 2011). The primary forests are dominated by mature trees, which can reach heights of up to 50 m (Caldecott & Miles, 2005). The main canopy is relatively closed and is highly concentrated in places with intertwined foliage and branches. However, areas of wetlands and swamps create large areas of canopy discontinuity. The understorey is mostly very open and typified by vertically orientated tree trunks and suspending lianas, which offer ample vertical pathways to the canopy from the forest floor (Head et al., 2011). These highly complex environments pose many challenges for gorillas. Both access to arboreal environments from the ground and movement within the trees requires an understanding of the properties of arboreal supports (which can deflect or break under their weight) and the ability to adapt locomotor behaviours accordingly (Brownlow et al., 2001; Grand, 1972; Povinelli & Cant, 1995; van Casteren et al., 2012). Understanding how they find solutions to the problems of travelling and feeding in trees, whilst mitigating the risk of falling, requires an integration of their ecology, morphology, and behaviour; an approach encapsulated in the ecomorphology framework (Bock & von Wahlert, 1965; Soligo & Smaers, 2016; Wainwright, 1991). In this study, we aim to clarify how western lowland gorillas interact with their natural environment by identifying the strategies they use to access different heights and move around the forest canopy, and whether these are influenced by body size and hand dimensions.

Much of what is currently known about how western lowland gorillas navigate complex arboreal environments has come from Remis (1995), who documented the positional behaviour, diet and activities of gorillas in the Central African Republic. Remis (1995) found that the gorillas spent most of their arboreal time climbing quadrupedally and walking. Observations of prolonged bouts of suspensory locomotion or gap‐crossing between trees were rare, as the gorillas tended to travel terrestrially to move between feeding sites. While this was the first data of its kind, Remis' findings were based on a relatively small data set (approximately 25 h of observations) of semi‐habituated gorillas, with most (80%) of observations taking place during the rainy season. Furthermore, before the standardisation of primate positional behaviour (Hunt et al., 1996), quantitative studies like Remis used broad classifications (six locomotor categories), which have since been extended (e.g. Thorpe & Crompton's classification of 57 biomechanically distinct modes of orangutan locomotion, 2005). Primates (including the great apes) cannot rely on a single or few locomotor behaviours because these must fulfil survival needs in complex environments. Therefore, to identify the key mechanical stressors that have influenced the evolution of their morphology, we need to understand comprehensively how they forage, avoid predators and maintain social relations in forests that are characterised by complex structures.

In comparison to the sparse data available for gorillas, the locomotion of orangutans in complex arboreal environments has been well studied (Cant, 1987; Manduell et al., 2012; Sugardjito & Van Hooff, 1986; Thorpe et al., 2009; Thorpe & Crompton, 2005) and has benefited from the development of detailed and uniform classifications of positional behaviour (Hunt et al., 1996; Thorpe & Crompton, 2005). Their locomotor repertoire is dominated by suspensory and forelimb‐balanced behaviours and is shaped by ecological factors such as branch diameter, type of supports and number of supports used (Thorpe & Crompton, 2005). Height in the canopy and context of behaviour were also found to be highly influential in determining their locomotor strategies (Thorpe & Crompton, 2005). In contrast, gorillas routinely exploit terrestrial environments and yet must find solutions to the same arboreal challenges despite conflicting demands on their musculoskeletal system (Doran, 1996; Remis, 1995). The evolutionary selective pressures on gorillas have favoured large size (because of sexual selection) (Cassini, 2020; Leigh, 1995; Pickford, 1986) as well as adaptations for terrestrial walking to enhance their efficiency when travelling on the ground. These include tightly packed carpals (Kivell & Schmitt, 2009), broad pedal bones to provide a stable weight‐bearing area during touchdown and short, robust manual bones also to increase stability during compression (Gebo, 1992; Schultz, 1963). Whether these traits have compromised their success or energetic efficiency in the trees requires a detailed examination of their locomotor ecology.

Larger body size is associated with higher absolute cost during locomotion because large primates have less muscle force per unit mass (Fleagle, 1985). Minimising energy expenditure is crucial for their survival as they need to retain sufficient reserves for critical activities such as feeding, social interactions, locomotion and predator avoidance (Arnold, 1983; Bock, 1994; Bock & von Wahlert, 1985; Wainwright, 1991). However, energy requirements and expenditure are different for gorillas of different body sizes. As body weight increases, so does metabolic rate, but the energy required per unit body weight decreases; this means that smaller gorillas have relatively higher energetic and nutritional requirements than larger gorillas (Jungers, 1984; McNab, 1978). Locomotion is therefore more energetically demanding for small gorillas on a per unit‐body‐weight basis. Further, females experience higher nutritional requirements (relative to body size) because of reproduction, as gestation, lactation and carrying dependent infants are metabolically costly (see, e.g. Lee et al., 1991; Nowell & Fletcher, 2008; Pontzer et al., 2014; Ross, 2001; Thompson, 2013; Wall‐Scheffler et al., 2007). Adolescents also require a high intake of calories to support physiological demands during growth. In arboreal environments, gorillas must strike a balance between their energy efficiency and mitigating the risk of falling, which varies according to age–sex and body size differences. Although Remis (1995) broadly discussed the variation in locomotor behaviours, support use, and tree location between female and male gorillas, the influence that body size has on their arboreal locomotion is still not fully understood. We predict that for the gorillas in Loango, body size differences will be associated with variation in arboreal behaviours and that the silverback gorilla might be restricted to less‐risky behaviours and locomotion on large supports because of his size.

The way in which gorillas address the ecological challenges of accessing different heights, travelling around the canopy, and dealing with discontinuity requires not only an understanding of their locomotor behaviours, but also of the arboreal supports that they use. Small, compliant supports can be riskier as they may be more likely to break under the weight of gorillas, but large supports are not always present in the periphery of tree crowns where nutritional resources (fruit and leaves) are often located. Therefore, how are the largest arboreal apes, the gorillas, able to forage and feed in arboreal environments? Orangutans, which are almost exclusively arboreal, are able to suspend from small supports when travelling and feeding in the trees (Thorpe & Crompton, 2005). Their behaviour follows the classic prediction that, for larger primates, suspending beneath a small support is less risky than travelling on top of it as the individual has effectively already fallen‐off the support (Cartmill & Milton, 1977). However, understanding whether gorillas can resort to suspension in the same way as orangutans can, or whether they are more restricted to larger supports, is a key component of determining their ability to exploit arboreal resources.

One of the biggest biomechanical challenges when moving in the trees is gap‐crossing. Crossing gaps requires moving across empty space within or between trees and is accompanied by considerable risk for all primates (Graham & Socha, 2020). Compared to walking and climbing, gap‐crossing demands a considerable number of skills. This includes an accurate judgement of distances, an understanding of physical properties, such as branch compliance, the ability to reach and grasp supports accurately and the musculoskeletal mechanisms to deal with support instability (Cartmill & Milton, 1977; Druelle et al., 2020; Fleagle et al., 1981; Graham & Socha, 2020; Larson & Stern Jr., 2006; Susman, 1984). In addition, gap‐crossing can be highly energetically demanding as compliant supports can require increased muscular effort and metabolic cost (Graham & Socha, 2020; Schmitt, 1999). Orangutans are highly competent at gap‐crossing not only because of physiological adaptations, but also because of their cognitive and physical ability to manipulate branches and lianas to ‘sway’, ‘ride’ and bridge between supports (Thorpe & Crompton, 2006; Thorpe, Crompton, & Alexander, 2007). In fact, gap‐crossing using compliant supports is significantly less costly for orangutans than jumping or descending and reascending a tree, as they can utilise energy stored in flexible branches during movement to their advantage (Thorpe et al., 2009; Thorpe, Crompton, & Alexander, 2007). It might be expected that gorillas will be exposed to greater risks when crossing gaps in the trees, given their larger size. However, since vertical climbing also requires significant muscular effort (Fleagle, 1985; Hanna et al., 2017; Hanna & Schmitt, 2011; Isler, 2005; Pontzer & Wrangham, 2004), it is possible that gorillas opt for risky gap crossings rather than descending to the ground and reascending trees to reduce the energetic cost of locomotion (Hunt, 1991). While there is some data on comparative energetic costs for locomotor behaviours (e.g., Thorpe, Crompton, & Alexander, 2007), we have not yet quantified the energetic demands of arboreal locomotor behaviours in primates. Given their size and morphology, we predict that gorillas have to balance the cost of arboreal locomotion with the risks required to achieve the goal of feeding in the trees.

What may also influence their success in arboreal environments is their hand morphology (specifically their hand proportions) and the postures used by gorillas. Gorillas have the highest thumb‐forefinger index of the great apes, which is more similar to that of humans than to that of other great apes (Almécija et al., 2015; Schmitt et al., 2016; Schultz, 1930). Orangutans are able to grasp multiple branches and lianas at once because of their long digits (Thorpe & Crompton, 2005), but gorillas may be restricted in the number of supports that they can hold because of their hand dimensions. Gorillas therefore may have to adjust their behaviour in arboreal environments to compensate for the adaptations they have in their hands for efficient terrestrial quadrupedal walking (Gebo, 1992; Schultz, 1930, 1963; Tuttle, 1967). Although the biomechanics of hand postures during quadrupedal walking have received much attention from a morphological perspective, there is a notable gap in our understanding of arboreal hand postures for wild western lowland gorillas (Kivell & Schmitt, 2009; Neufuss et al., 2017; Tarrega‐Saunders et al., 2021). ‘Knuckle‐walking’ dominates the hand postures used during the terrestrial quadrupedalism of mountain gorillas (Thompson et al., 2018). However, in arboreal settings, palmigrady has been observed as a common hand posture, which increases contact area and thus can adjust for stability when moving on top of compliant or smaller supports (Finestone et al., 2018; Hunt, 1992a; Kivell & Schmitt, 2009; Preuschoft, 2002; Tarrega‐Saunders et al., 2021). There is currently very little data on the hand postures of gorillas when engaging in different locomotor behaviours on a variety of support types. In a study of vertical climbing in mountain gorillas, Neufuss et al. (2017) found that grip type depended on the size of the support. Larger supports (>10 cm in diameter) were grasped using power grips (without the use of an opposing thumb), whereas smaller supports were grasped using diagonal power grips (aided by thumb opposition) (Napier, 1960; Neufuss et al., 2017). This indicates a potential relationship between support type, locomotion and hand postures, which we aim to investigate further by examining the role of hand posture in the arboreal locomotor ecology of wild gorillas. This may reveal how adaptations towards terrestrial locomotion may restrict the gorillas' ability to access different supports in arboreal environments which are characterised by considerable support variability.

The aim of the present study is to identify the ecological, morphological and behavioural aspects that shape the arboreal locomotor ecology of western lowland gorillas. In order to investigate whether their adaptations towards terrestrial quadrupedal walking and the great range in body size influence their exploitation of arboreal environments, we aim to address several questions: (i) how do the gorillas in Loango access and move around the canopy to feed on arboreal resources, (ii) what strategies do they use to deal with discontinuity, (iii) is risk mitigated by using specific supports, especially at higher heights, (iv) does hand dimension influence support use, (v) are classic body size predictions supported by variation in locomotor behaviours and support use between the silverback, adult females and adolescents? By addressing these questions and thus developing our knowledge of the form‐function interface of our closest living relatives in their natural habitats, we can develop our understanding of the relationship between form and behaviour in ancestral apes.

## 2 METHODS

### 2.1 Location

**FIGURE 1:**
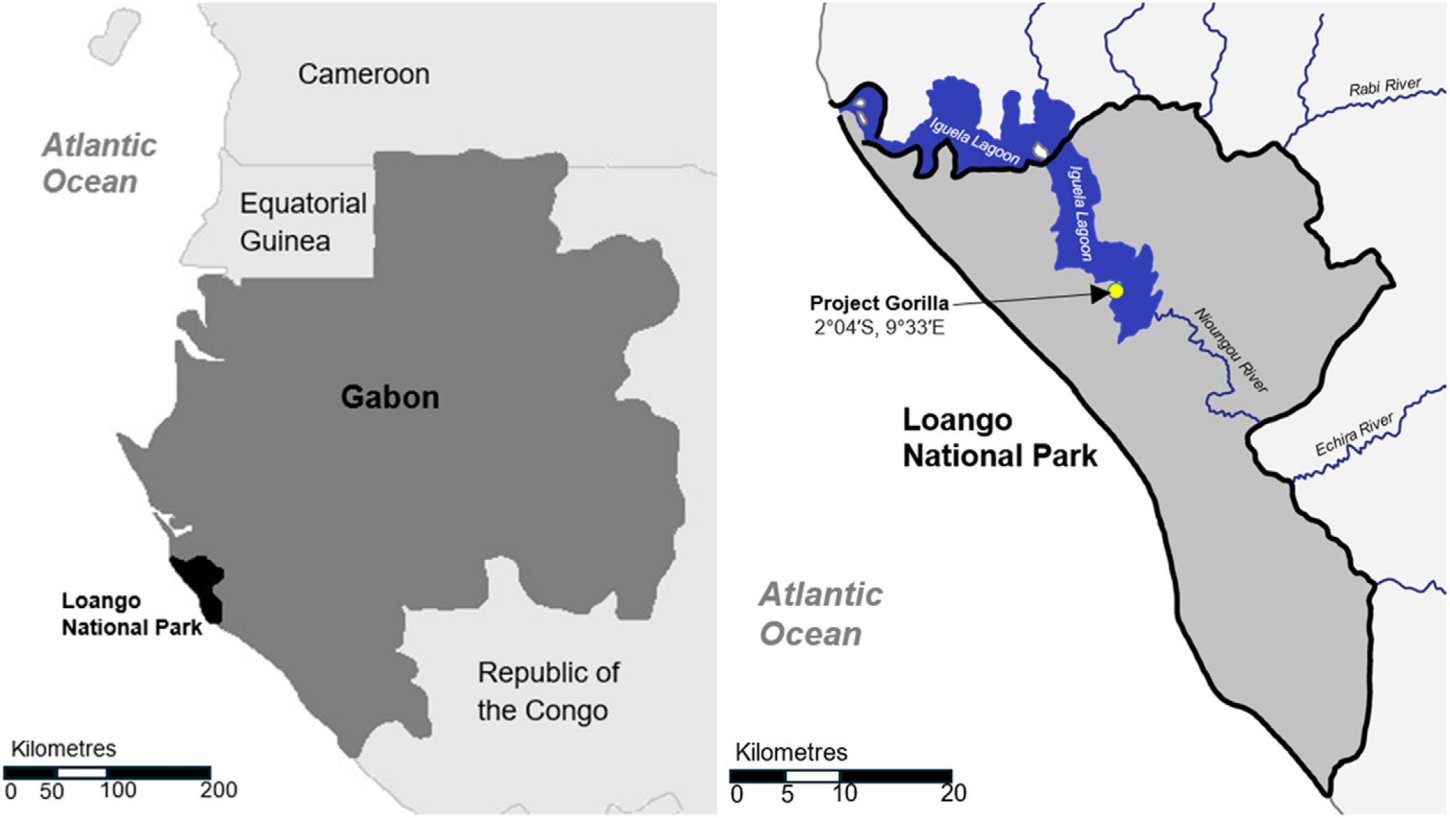
Location of the Loango National Park in Gabon, showing the position of the lagoon and research site, adapted from Harris et al. (2012).

**FIGURE 2:**
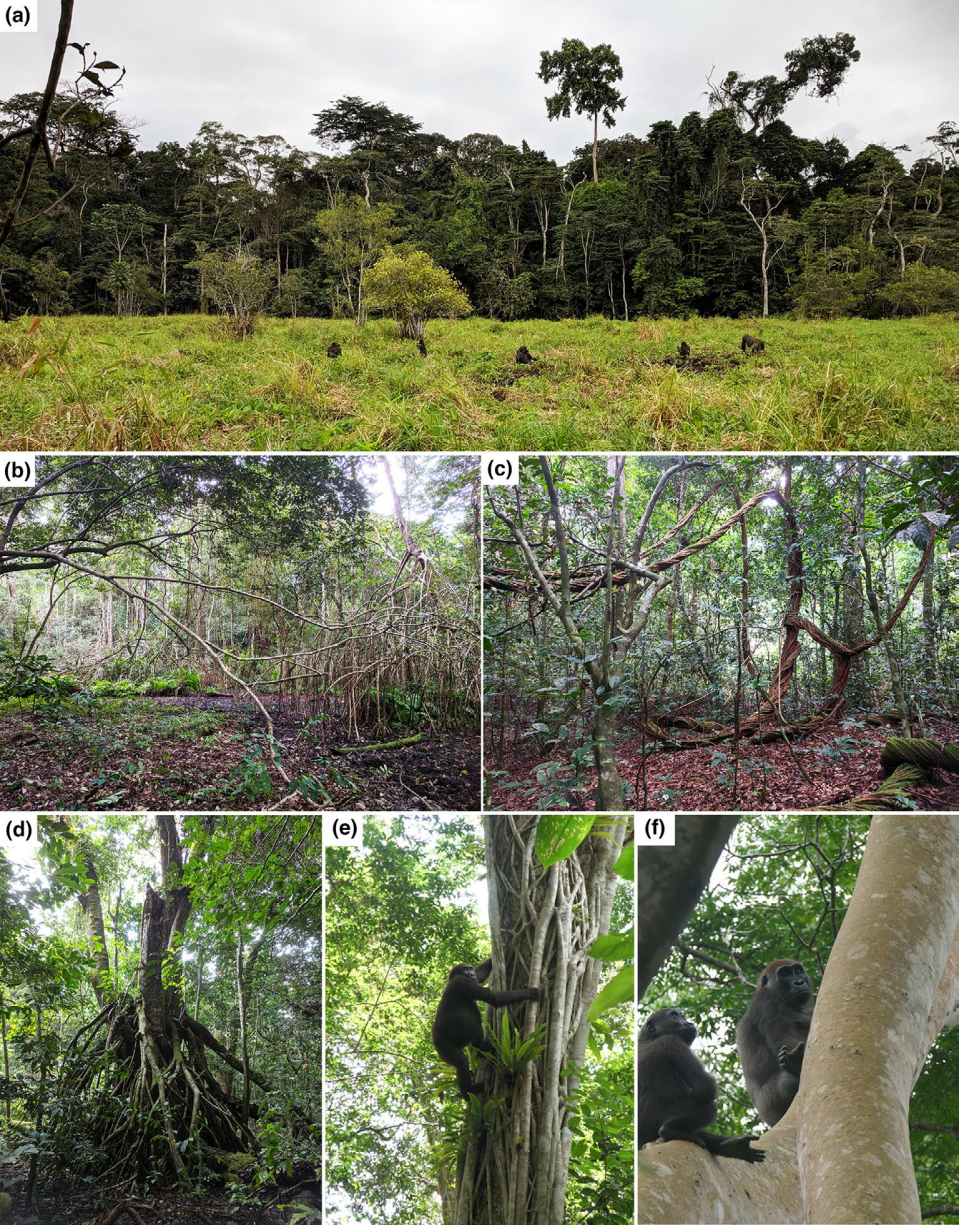
Images of the habitats used by the gorillas in the Loango National Park. Image (a) shows a large swamp typical of Loango, which is marked along the border by mature trees and a lack of tall trees within the swamp. Along the border of the lagoon, mangrove forests create intertwined networks which vary seasonally in height above the water, as seen in Image (b) which was taken during the dry season. Image (c) shows a network of suspending lianas that vary in size and orientation. Image (d) is a *Uapaca guineensis*, and images (e) and (f) are of gorillas in *ficus* sp., some of the larger fruiting trees.

The field study took place between July 2021 and July 2022 at the Loango Gorilla Project of the Max Planck Institute of Evolutionary Anthropology in the Loango National Park, Ogooue‐Maritime, Gabon (2°04′ S and 9°33′ E). The study site is on a long strip of land bordered to the west by the Atlantic Ocean (Figure 1) and mostly comprises primary forest and lowland swamps fed by the lagoon (Figure 2) (Head et al., 2011). There is a long rainy season typically between October and April, which is often interrupted by a short dry season from December to January (Head et al., 2011). The annual temperature in Loango ranged between 18 and 28° (Hagemann et al., 2019; Klein et al., 2021).

### 2.2 Subjects

The study group consisted of nine habituated wild western lowland gorillas, including one silverback, three adult females, one blackback (typically between the age of 11 and 14 years for western lowland gorillas), one subadult (between the age of 7.5 and 10 years for males), one juvenile (between the age of 4 and 7.5 years) and three dependent infants (below the age of 4 years) (Table 1; age ranges described by Breuer et al., 2009). In this study, positional behaviour was not collected for infant individuals under the age of two because of their dependency on being carried by their mothers (LaRocque, 2009; Nowell & Fletcher, 2008). Body weight was estimated using a combination of comparisons with known weights of captive gorillas and typical weight ranges documented for wild individuals. While captive gorillas are generally larger than their wild counterparts, their reliably documented weights provided a baseline for estimation (Leigh, 1994; Zihlman & McFarland, 2000). We verified our estimates against typical weight ranges for wild western lowland gorillas, with adult males averaging around 170 kg and females around 70–75 kg (Jungers & Susman, 1984). The absolute weight of adolescent gorillas is not fully documented, as it varies greatly with age and developmental stage; therefore, we estimated their weights relative to their size in comparison to adult females. However, by grouping individuals into body size categories based on age and sex classes, we focused on relative differences between groups rather than relying on absolute weight estimates (Table 1).

**TABLE 1:** Sex, age and estimated body weight of the seven gorillas which made up the study subjects of this research.

Individual	Sex	Age^a^	Estimated body weight (kg)	Notes
Kamaya	Male	30–35	~170	Silverback, dominant male
Tonda	Female	25	80	Adult female, gave birth to infant 25.03.22 and mother of Ogwely
Ambia	Female	25	75	Adult female, mother of Waka and dependant infant Malumbi (born 26.08.20)
Mokebo	Female	30	70	Adult female, mother of Orema and dependant infant who was born on 23.05.21, and died 26.06.22
Waka	Male	11	75	Blackback, offspring of Ambia
Orema	Male	9	35	Subadult, son of Mokebo, independent
Ogwely	Male	5	30	Juvenile son of Tonda, independent

^a^ Age from the beginning of the study.The exact age of Orema and Ogwely is known, but for other individuals, age estimation is based on body size descriptions in Breuer et al. (2009) and knowledge of gorilla size and age.

Focal individuals were chosen at random each morning prior to locating the group and were followed for the duration of 1 day. First contact was made after 7:00, once the group was located, between 7:05 and 12:00 (*n* = 263 days) with few occasions where contact was not made for the duration of the day. The gorillas were followed until 16:30. If the focal animal moved out of sight for more than 2 h, another individual was selected at random and observed for the remainder of the day (*n* = 5 days). The gorillas would frequently traverse mangrove thickets and swamps that were not suitable for the research team to follow. In this case, the contact would be paused until the focal animal was seen again. When high in the canopy and positioned in areas of dense vegetation, identification of the focal individual and other behavioural and ecological data would also often be difficult to observe, so partial observations were recorded such as height‐only or locomotor data without information on support use. Data were collected throughout the duration of the study by a single observer. Considerable self‐training and regular testing was performed to maintain accuracy in estimating support properties and height using a laser rangefinder.

Instantaneous data were collected on the 1‐min mark (Altmann, 1974; Doran, 1992; Thorpe & Crompton, 2005). Every minute, locomotor behaviour, height and hand posture were documented, as well as support properties for each weight‐bearing limb (Table 2).

**TABLE 2:** List of locomotor ecology variables collected in the field using 1‐min instantaneous sampling.

1.	Date
2.	Time
3.	Individual: Kamaya, Tonda, Ambia, Mokebo, Waka, Ogwely, Orema
4.	Weather: wet, when raining and dry when not raining (recorded for each data point)
5.	Locomotor mode and submode: 15 modes with 57 subsequent submodes^a^ Modes: Quadrupedal walk, tripedal walk, bipedal walk, vertical climb, vertical descent, torso‐orthograde suspension, torso‐pronograde suspension, forelimb‐hindlimb swing, bridge, leap, sway and ride
6.	Height (m): 0–2, 2–5, 5–10, 10–15, 15–20, 20–25, 25–30, >30 (defined as height from ground directly below the focal individual)
7.	Support type: tree trunk, branch, liana, liana/branch (combination of tree branch and liana), branch bundles (clusters of small branches intertwined and used in bunches)
8.	Support diameter: 0–4 cm, 4–10 cm, 10–20 cm, 20–40 cm, 40+ cm (recorded for each weight bearing limb)
9.	Support orientation: horizontal (0° ± 20°), angled ~45° (and supports between >20° of horizontal and <20° of vertical), vertical (90° ± 20°), U‐shaped, mixed orientations (for each weight bearing limb)
10.	Number of limbs in contact with supports: 1, 2, 3 or 4 for each weight bearing component, defined as a limb or body part that is bearing more weight than the limb itself (Hunt et al., 1996)
11.	Number of supports: 1, 2–4, >4 (Thorpe & Crompton, 2005)
12.	Behavioural context: travelling; feeding (actively seeking, accessing, processing and consuming food); other (resting; grooming; grooming other; affiliative (Pereira & Altmann, 1985); playing; agonistic threaten; agonistic attack (Harcourt, 1979); sexual (Stoinski et al., 2009); avoid (Pereira & Altmann, 1985); autoplay; chest beat; nest building; vigilant (Kutsukake, 2007); breastfeeding; human watch (where focus is on researchers‐often as an intense stare that lasts several seconds))
13.	Infant position: dorsal, ventral without forelimb support, ventral with forelimb support, arm, head, parked (deposited by mother on the ground or in the trees on a support)
14.	Hand position: for tensile postures, power grip (all five digits and the entire palm in contact with the support), diagonal power grip (small supports rest diagonally across the fingers and palm), diagonal finger hook grip (the thumb and palm is not used, but instead supports will rest on the phalanges) (Napier, 1960). For compressive postures: knuckle, fist, palm, wrist, forearm (Thompson et al., 2018)

^a^ Submodes defined in Appendix S1.

### 2.3 Data analysis

The aim of the statistical analysis was to explore whether there were relationships among locomotor behaviours, height, support use and hand postures and to ascertain whether these varied based on body size. A methodological data exploration protocol was established to address the complexities in the data set. This approach involved appropriate conflation of variables and descriptive analysis using bivariate standardised cell residuals to examine two‐way relationships. Subsequently, statistical techniques were used to expose underlying patterns in the data.

In order to enhance model interpretability and avoid issues of overfitting and zero‐inflation (as a consequence of too many combinations of variables), variables were conflated (see Table 3). Locomotor behaviours were grouped based on biomechanical similarities; for example, the exploitation of specific muscle groups or the loading of specific anatomical structures depending on the orientation and contact of specific limbs and the external forces acting on a body part influencing motion. To examine the influence of body size, individuals were combined into appropriate body size groups based on body weight estimations. Height was also conflated, for statistical analysis, into three arboreal categories that represent the different layers in the forest: <10, 10–20, and >20 m. Although there is variation because of the differential maturity and species of trees, these categories reflected the general stratification of the forest. It was found that the canopy typically began to develop at heights of around 10 m, but at heights above 20 m, it was less dense and sparser; these categories were therefore deemed to be ecologically meaningful. For each of the gorilla's weight‐bearing limbs, data were collected for the support type, diameter and orientation that it contacted. This resulted in a number of combinations too large to include in a multivariate analysis, so the data were combined. Support diameter was grouped into five categories: small, medium, large, branch bundles and mixed sizes. Support orientation was grouped into five categories: vertical, angled, horizontal, U‐shaped and mixed orientations. Behaviour was split into ‘travelling’ and ‘feeding’ with other behaviours removed because of the small number of observations (*n* = 31). Feeding was defined as actively seeking, accessing and consuming food.

**TABLE 3:** Description of conflated categories for locomotion, body size, arboreal height and support parameters used within the modelling process.

Variable	Conflated variables	Description
Locomotion	Walking	Quadrupedal walk, Tripedal walk, Bipedal walk
	Vertical climbing	Vertical climb, Vertical descent
	Suspension	Torso‐orthograde suspensory, Torso‐pronograde suspensory, Forelimb/Hindlimb swing
	Gap crossing	Bridge, leap, sway, ride
Body size (kg)	170	Adult male (silverback)
	~70	Adult females, blackback
	<40	Subadult, juvenile (hereafter known as adolescents)
Height (m)	<10	Understorey
	10–20	Low and main canopy
	>20	Main and emergent canopy
Support diameter	Small	All supports ≤10 cm
	Medium	All supports 10–20 cm
	Large	All supports >20 cm
	Mixed	Mixture of diameters
	Bundles	All supports are branch bundles
Support orientation	Vertical	Vertical: 90° (±20°)
	Angled	Between >20° of horizontal and <20° of vertical
	Horizontal	Horizontal: 0° (±20°)
	U‐shaped	U‐shaped lianas
	Mixed	Combination of different orientations
Behaviour	Travelling	Travelling is the primary behaviour used to move between feeding sites
	Feeding	Specific movement patterns while actively seeking, accessing and consuming food

### 2.4 Statistical analysis

Statistical analysis was conducted in R version 4.3.1 (2023) with the following packages: *lme4* (version 1.1–34, Bates et al., 2014) for fitting generalised linear mixed models and *AICcmodavg* (version 2.3–2, Mazerolle, 2019) for model selection using AIC. Graphs were produced using the *ggplot2* package (v3.3.3; Wickham & Wickham, 2016).

Observations were transformed into count data to represent the frequency of occurrences. As such, generalised linear modelling (GLM) was chosen as the most appropriate model for data analysis, using the Poisson log‐link function, the most suitable method for count data. A Poisson distribution ([g(μ) = log(μ)], where g(μ) is the link function and μ is the mean of the Poisson distribution) was used for response variables in all models (Nelder & Wedderburn, 1972; Zuur et al., 2009). These models provided a suitable framework for examining the occurrences of events, such as combinations of locomotor ecology variables. The rate parameter, or lambda (λ), was used in the models to represent the expected number of events for a given combination of predictor variables.

For the fitted Poisson GLM models, the Akaike's Information Criterion (AIC) was used to identify which model interactions best represented the data (Aho et al., 2014; Bozdogan, 1987; Van Andel et al., 2015). In order to deal with overfitting, the AIC uses the measure of parsimony by adding penalties for additional predictor variables within models of higher complexity, so that models with more variables are not preferred over models with fewer variables (Aho et al., 2014). Backwards stepwise regression was performed on the models using AIC as the selection criteria to determine which variables contributed significantly to the fit of the data. This process eliminated variables that did not increase the fit of the model, leaving only interactions and main effects that played a substantial role in the observed patterns in the data.

To test for potential heterogeneity, several generalised linear mixed models with random effects were performed with individual specified as a random effect to assess whether individual variance should be accounted for in the final models (Bolker et al., 2009; Zhu & Zhang, 2006). However, there was not a significant improvement in the models' fit to the data when accounting for potential unobserved variability due to individual identity. Any benefit gained from the inclusion of individual variation was offset by model complexity, meaning that the GLM's provides a better balance between model fit and complexity. The Poisson regression models provided statistical estimates of the expected counts of particular variables and interactions in order to make comparisons between different groups. To avoid problems of multicollinearity, separate models were performed differentiating between variables that display high correlation: this ensured that associations between variables were not influenced by the effects of collinearity, thus improving the interpretability of results (Dormann et al., 2013).

Diagnostic tests were performed to ensure that the models satisfied the assumptions of Poisson GLM analysis. This included a systematic examination of linearity, independence, homoscedasticity and zero‐inflation. Zero inflation is a common issue in ecological data sets where an excess number of zeros is observed compared with an expected count distribution. This occurs as a result of rare events that are documented in small frequencies and are included in models with other ecological variables that occur in large frequencies. The GLM final models were tested for zero‐inflation using the *Performance* package (version 0.10.4, Lüdecke et al., 2021) and the models revealed a maximum ratio of 1.52 (observed zeros: predicted zeros) indicating a suitable amount of observed zeros (Deng & Paul, 2005; Gurmu, 1991).

Post hoc analysis was conducted to assess the strength of variables within the models using standardised cell residuals (SCRs) of multiway contingency tables. These identified discrepancies between observed and expected values which exceed expectations, revealing underlying correlations within a significant interaction (Beasley & Schumacker, 1995). SCRs of absolute values above 2.0 and below −2.0 contribute to the overall statistical significance (Haberman, 1973). SCRs above 2.0 suggest that an interaction was documented more than predicted, whereas below −2.0, the interaction occurred less than predicted by the model.

### 2.5 Limitations

GLM models using AIC as the selection criteria are valuable tools in model selection. However, model selection is not straightforward and there is no universal method for selecting the ‘best’ model (Brewer et al., 2016; Burnham & Anderson, 2004). In ecological literature, the most appropriate approach for AIC use within stepwise regression involves an integration of balanced statistical metrics and an understanding of the biological relevance of a model. The aim was to strike a balance between model complexity and capturing underlying patterns in the data. There was only one silverback in the largest body size category, so conclusions based on body size should be interpreted with caution.

## 3 RESULTS

Overall, 944 h of data and 56,635 instantaneous positional behaviour observations were collected (Table 4). Of these, the focal gorilla was visible for 35,951 observations (599 h). Height only observations (when the focal location was known, but not visible) accounted for 193 h. The silverback was visible the most, whereas the blackback was visible the least. The silverback was documented to move the least when off the ground, whereas the youngest individual moved the most.

**TABLE 4:** Total focal time, amount of time spent arboreally and quantity of time observed spent engaging in locomotion for each individual subject.

Individual	Age–sex class	Total focal time (h)	Total data points (*n*)	Out of sight (% of total observations)	Height only (*n*)	Total visible observations (*n*)^a^	Locomotion (% of total positional behaviour)	Arboreal locomotion (% of all arboreal positional behaviour)^b^	Arboreal locomotor observations (*n*)^c^
Kamaya	Silverback	118	7094	1109 (15.6)	1252	4733	16.3	7.7	90
Tonda	Adult female	136	8142	2954 (36.3)	1707	3481	17.7	9.6	187
Ambia	Adult female	145	8687	3134 (36.1)	1663	3890	19.3	10.5	206
Mokebo	Adult female	144	8667	3296 (38.0)	1953	3418	24.1	12.1	170
Waka	Blackback	122	7308	3685 (50.4)	1249	2374	23.2	11.7	150
Orema	Adolescent	140	8380	3109 (37.1)	1933	3338	22.3	11.8	235
Ogwely	Adolescent	139	8357	3397 (40.6)	1846	3114	27.0	14.8	300
Total		944	56,635	20,684 (36.5)	11,603	24,348	20.9	11.3	1338

^a^ While height‐only observations represent occasions where the height of the gorilla was known, but the focal was completely out of sight, ‘visible’ observations account for when the gorilla was visible in some way (either their whole body or body part).

^b^ Arboreal locomotion denotes how much time each individual gorilla engaged in locomotion when off the ground (when they were visible). For example, when off the ground, the youngest gorilla, ‘Ogwely’, engaged in locomotion the most.

^c^ Data used in the modelling process in this study.

**FIGURE 3:**
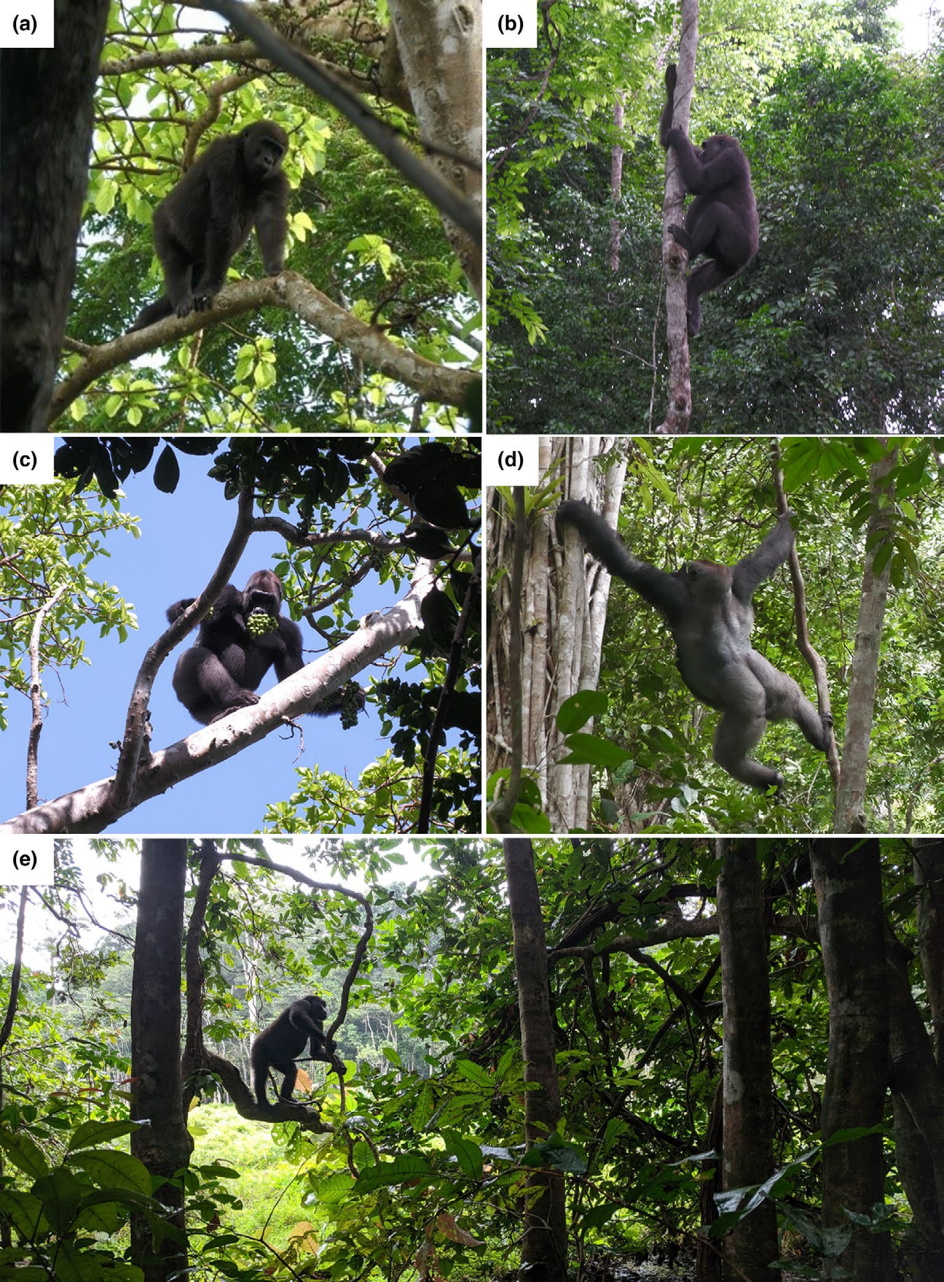
Screen captures of mid‐bout locomotion showing differences in locomotor behaviour (a) adolescent individual using quadrupedal walking; (b) adult female individual using flexed‐elbow vertical descent; (c) silverback engaging in hand‐assisted bipedal scrambling within the category of walking; (d) silverback using a forelimb‐swing, a torso‐orthograde suspensory locomotion, within the locomotion category of suspension; (e) blackback using hand‐assisted flexed bipedal walking.

Overall, the arboreal locomotion of the gorillas was dominated by walking (44%). Of these walking behaviours, quadrupedal walking was used the most, accounting for 67%, and bipedalism (including hand‐assisted) made up 31% (Table 5). Vertical climbing accounted for 36% of all arboreal locomotion. Suspension, which was almost exclusively torso‐orthograde, occurred in slightly higher frequencies than gap‐crossing (13% vs. 7% respectively). Twenty eight percent of all gap‐crossing behaviours were oscillatory, which included ‘ride’ and ‘tree‐sway’. Figure 3 shows some of the variation in locomotor behaviours used by the gorillas.

**TABLE 5:** Percentage of arboreal locomotor modes for all gorillas and each age–sex category.

Locomotor mode	All individuals	Silverback	Females + blackback	Adolescents
Quadrupedal walk	29.0	21.2	32.4	25.7
Tripedal walk	0.7	0	1.1	0.2
Bipedal walk	13.8	15.3	13.3	14.2
Vertical climb	16.2	20.0	15.4	16.7
Vertical descent	20.1	28.2	20.9	17.5
Torso‐orthograde suspension	12.6	8.2	11.3	15.0
Torso‐pronograde suspension	0.6	1.2	0.1	1.2
Forelimb‐hindlimb swing	0.2	0	0	0.4
Bridge	3.2	0	2.9	4.1
Leap	1.8	2.4	0.7	3.1
Sway	0.2	0	0.4	0
Ride	1.7	3.5	1.3	1.9
Number of arboreal locomotor observations	1296	86	697	514

Excluding vertical climbing (ascent and descent) which elicits orthograde trunk postures (Crompton et al., 2008) and mixed/unidentified torso orientations (for example during branch oscillation), when moving arboreally, the gorillas used torso‐pronograde locomotor modes marginally more, accounting for 54% of their arboreal locomotion. Torso‐orthograde locomotor modes made up 46% of their arboreal locomotor behaviours.

### 3.1 Arboreal locomotion

The initial model that examined the interaction between locomotion, height, behaviour and body size tested whether there were any significant relationships between the locomotor behaviours, contextual behaviours and heights used by the gorillas, and whether these varied between body size groups. The modelling process removed all three‐way interactions but retained all possible two‐way interactions (Table 6).

**TABLE 6:** Poisson GLM model results for the interaction between locomotion, body size, height and behaviour.

Model	Model variables	Retained interactions^a^	Deviance (*df*)	*p‐*Value	Residual deviance (*df*)^b^
1	Locomotion	Locomotion: body size	23.86 (6)	<0.001	67.71 (68)
	Body size	Locomotion: height	28.74 (6)	<0.001	
	Height	Locomotion: behaviour	83.90 (4)	<0.001	
	Behaviour	Body size: height	19.32 (4)	<0.001	
		Body size: behaviour	26.20 (4)	0.016	
		Height: behaviour	35.33 (4)	0.042	

Abbreviation: GLM, generalised linear mixed model.

^a^ All main effects are retained in the model.

^b^ Residual deviance and *df* for final model.

Overall, the gorillas moved in the trees at heights of <10 m 56% of the time. They used these lower heights for locomotion 1.5 times more than heights between 10 and 20 m and 5.9 times more than heights above 20 m. Locomotion below 20 m was dominated by walking and vertical climbing (Table 7). SCRs for the relationship between locomotion and height revealed that the gorillas walked at heights above 20 m more than predicted by the model (SCR = 3.5) but used vertical climbing at these heights less than the expected amount (SCR = ‐3.1). Suspension and gap‐crossing made up 5%–14% of locomotion for each height group and were documented within the expected range.

**TABLE 7:** Contingency table for the interaction between locomotion, and height, and locomotion and behaviour.

	Locomotion				
	Walking	Vertical climbing	Suspension	Gap‐crossing	Total
Height (m)					
<10	41.1 (54.3)	39.3 (62.3)	13.0 (56.1)	6.6 (41.2)	56.2
10–20	39.3 (32.6)	33.3 (33.2)	13.7 (37.0)	13.7 (53.8)	35.3
>20	65.5 (13.1)	18.6 (4.5)	10.6 (6.9)	5.3 (5.0)	8.5
Total	42.5	35.4	13.0	9.0	100
Behaviour					
Travelling	37.9 (73.1)	40.0 (92.3)	14.4 (90.4)	92.0 (92.0)	83.7
Feeding	71.6 (26.9)	17.2 (7.7)	7.8 (9.6)	8.0 (8.0)	16.3
Total	43.4	36.3	13.3	7.0	100

*Note*: Figures represent the row % and (column %) for each interaction. For example, the gorillas engaged in walking 41.1% of the time when moving at heights of <10 m, but 54.3% of the time that they were walking, they were at heights of <10 m. White cells signify that the SCR of the interaction was within the predicted range by the model. Coloured cells indicate whether the interaction fell below or above the predicted range (see key). 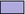, SCR > +2; 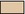, SCR < −2.

Abbreviation: SCR, standardised cell residuals.

The gorillas travelled 5.5 times more than they fed (actively searching and acquiring food) when engaging in arboreal locomotion. When feeding, the gorillas walked almost 70% of the time, in frequencies much more than predicted by the model (SCR = 6.1), whereas vertical climbing and suspension when feeding were observed in much lower frequencies than predicted (SCR = −4.5) (Table 7). When travelling, walking and vertical climbing each accounted for 36%–38% of locomotion, but while walking occurred less than expected (SCR = −2.7), vertical climbing was used in frequencies more than expected (SCR = 2).

As height increased, so did the amount of time that the gorillas spent feeding, but frequencies did not deviate from predictions based on SCRs. The gorillas used locomotion during feeding 12% of the time below 10 m, 19% of the time at heights 10–20 m, but 35% of the time above 20 m. The modelling process removed the interaction between height, behaviour and locomotion; this suggests that the locomotor behaviours used at different heights were not dependent upon whether the gorillas were travelling or feeding.

### 3.2 Body size

**FIGURE 4:**
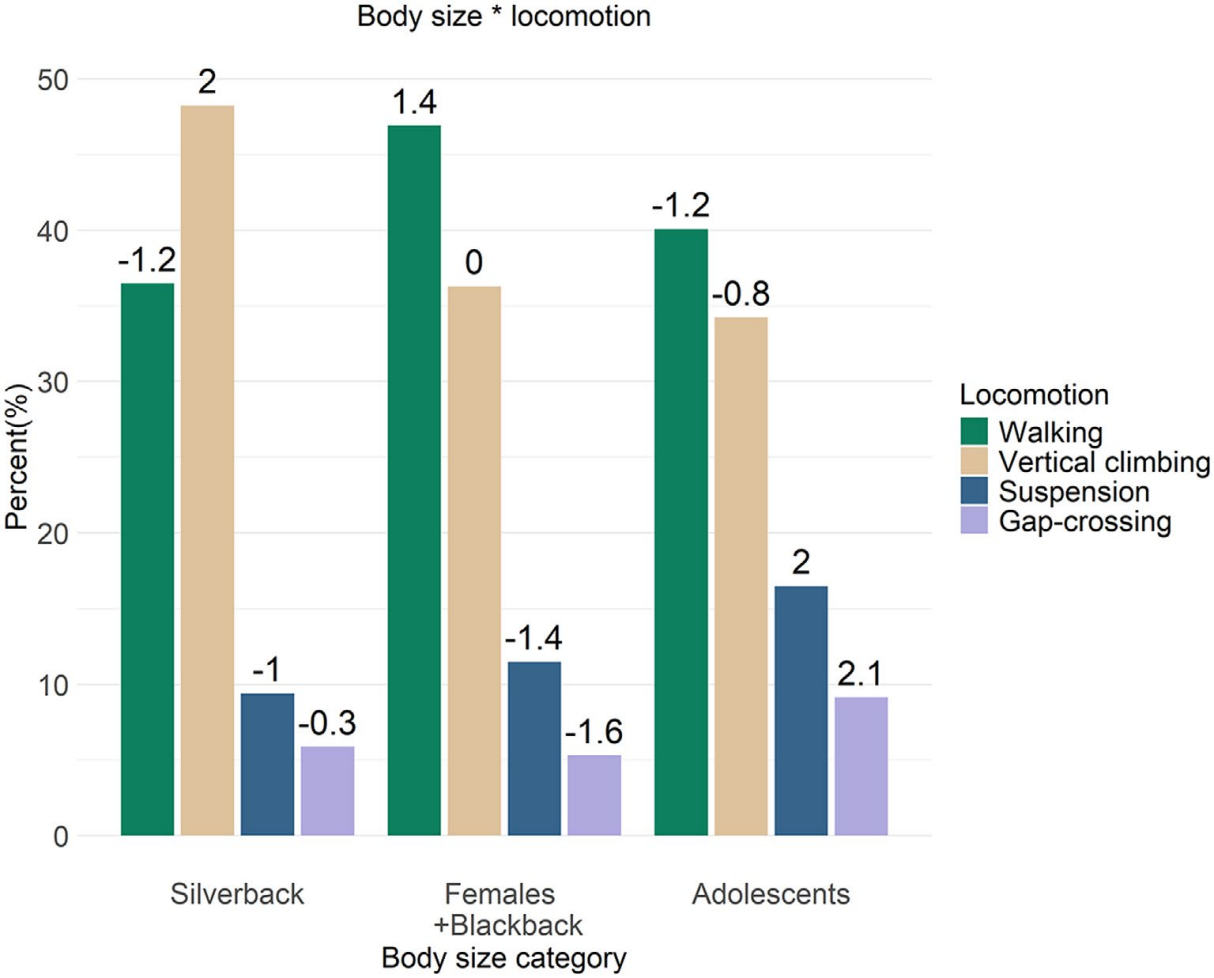
Model interaction between body size and locomotion when arboreal. The values above the bars represent the standardised cell residuals (SCRs).

The large degree of sexual dimorphism was reflected in the variation of frequency of locomotor behaviours, contextual behaviours and heights used by different body size gorillas. The silverback engaged mostly in vertical climbing (accounting for 46% of his locomotion), whereas the females and blackback used walking the most (also accounting for 46% of their locomotion) (Figure 4). The adolescents used walking and vertical climbing in similar relative proportions to the adult females and blackback, but with slightly lower frequencies. They did, however, engage in suspension and gap‐crossing more than the larger body size categories, in frequencies higher than predicted (SCR = 2 and 2.1, respectively). While the silverback exhibited a marginally higher propensity to engage in gap‐crossing than the females and blackback, the females and blackback used suspension more than the silverback.

**FIGURE 5:**
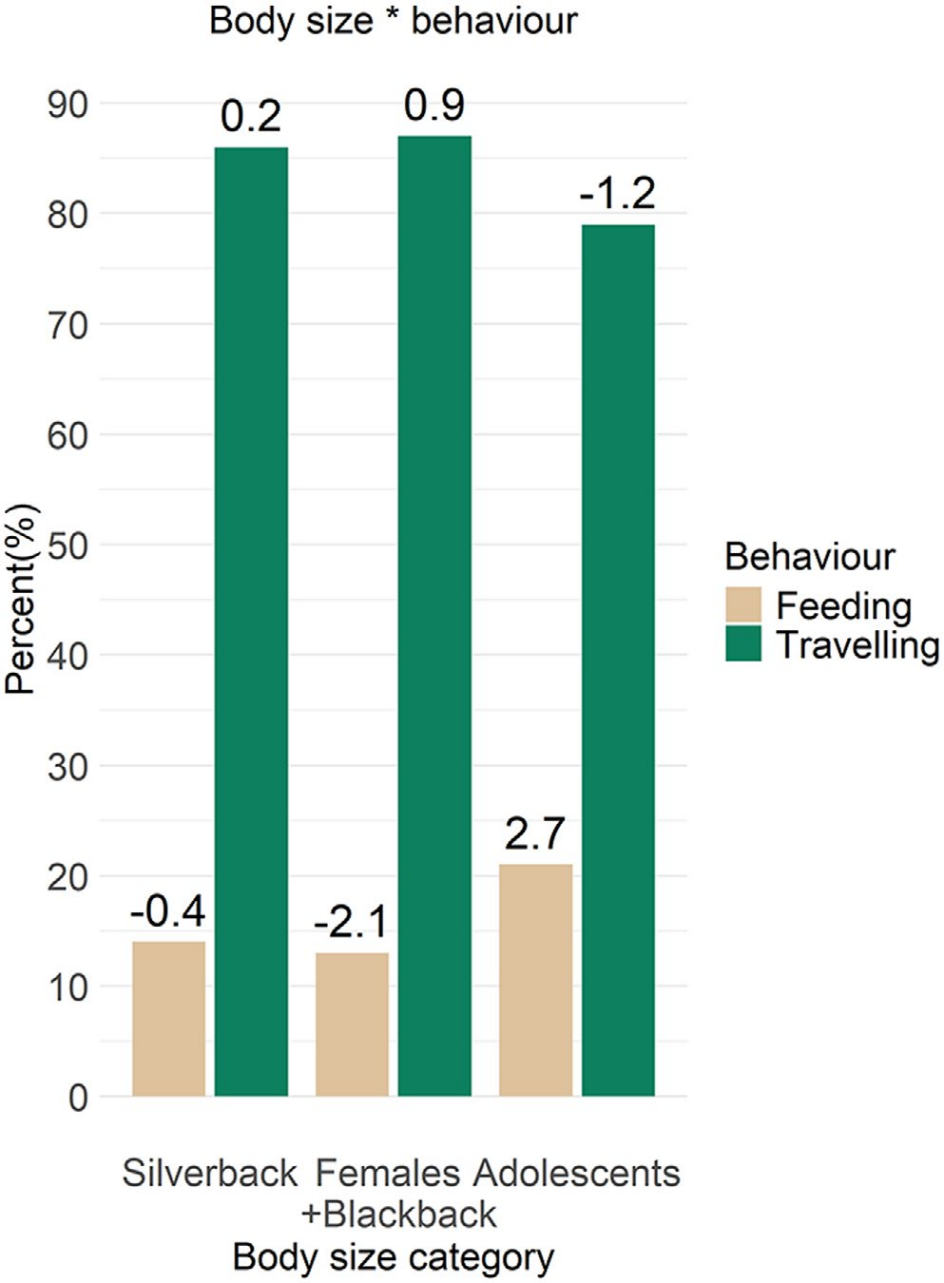
Model interaction between body size and behaviour when engaging in arboreal locomotion. The values above the bars represent the standardised cell residuals (SCRs).

The silverback, females and blackback locomoted during feeding 13–14% of the time, but while this fell within the predicted range for the silverback (SCR = ‐0.4), it was much less than predicted for the females and blackback (SCR = ‐2.1) (Figure 5). The adolescents used locomotion while feeding 20% of the time, more than expected (SCR = 2.7). Travelling for all body size groups did not deviate from the expected range based on SCRs.

**FIGURE 6:**
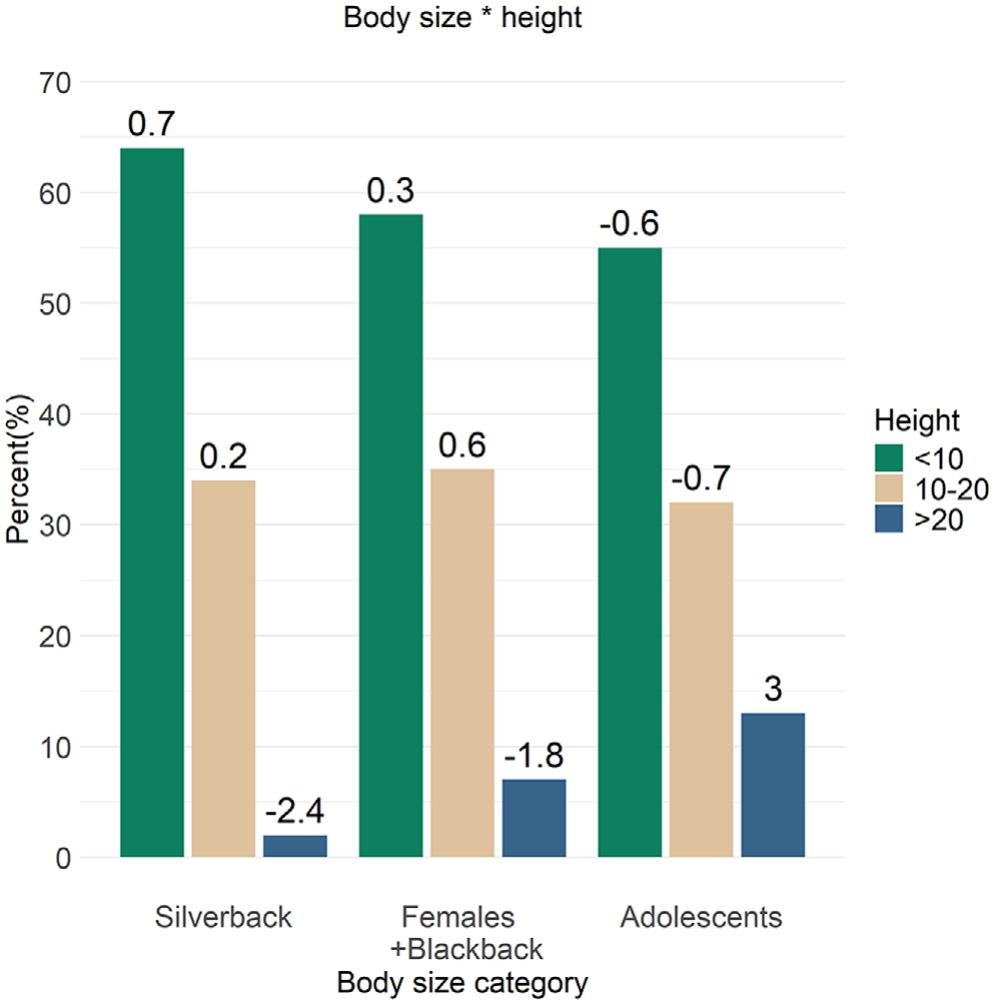
Model interaction between body size and height when engaging in arboreal locomotion. The values above the bars represent the standardised cell residuals (SCRs).

All body size categories spent the most amount of time locomoting below 10 m (56%–63%), followed by mid‐heights (31%–34%), all within the predicted SCR range (Figure 6). The silverback spent the least amount of time moving above 20 m, accounting for only 2%, which was a frequency much smaller than predicted by the model (SCR = −2.4). The females spent more time at this height (7%) and in frequencies as expected, but the adolescents used these heights much more than predicted (13%, SCR = 3.0).

### 3.3 Arboreal support use

Statistical Model 2 in Table 8 tested whether the supports that the gorillas used differed according to locomotor behaviour and if larger individuals compensated for their size by using different supports to smaller individuals. Locomotor behaviour was significantly associated with variation in support type, support diameter and support orientation. The removal of all interactions between body size and other variables except for diameter indicates that body size was accompanied by variation in the size of supports used, but not the type of supports, support orientation or the number of supports used.

**TABLE 8:** Poisson GLM model results for the interactions between locomotion and body size with support properties (Model 2), number of supports (Model 3) and hand posture (Model 4).

Model	Model variables	Retained interactions^a^	Removed interactions^b^	Deviance (*df*)	*p‐*Value	Residual deviance (*df*)^c^
2	Locomotion	Body size: diameter		22.28 (8)	<0.001	318.1 (644)
	Body size	Locomotion: diameter		157.87 (12)	<0.001	
	Diameter^d^	Locomotion: support type		83.55 (6)	<0.001	
	Orientation^d^	Locomotion: orientation		774.78 (9)	<0.001	
	Support type	Diameter: support type		128.4 (8)	<0.001	
		Diameter: orientation		202.8 (12)	<0.001	
		Orientation: support type		62.68 (6)	<0.001	
			Body size: locomotion	16.99 (6)	0.01	
			Body size: orientation	8.43 (6)	0.21	
			Body size: support type	1.66 (4)	0.79	
3	Body size	Body size: locomotion		16.99 (6)	<0.001	83.52 (72)
	No. of supports	Body size: height		9.60 (4)	0.048	
	Locomotion	Locomotion: height		26.51 (6)	<0.001	
	Height	No. supports: locomotion		59.79 (6)	<0.001	
		No. supports: height		11.84 (4)	0.019	
			No. supports: body size	3.25 (4)	0.516	
4	Body size	Body size: diameter		15.87 (8)	0.04	140.37 (324)
	Locomotion	Locomotion: diameter		143.75 (12)	<0.001	
	Diameter	Locomotion: hand posture		538.31 (18)	<0.001	
	Hand posture	Diameter: hand posture		575.15 (24)	<0.001	
			Body size: hand posture	23.66 (12)	0.02	
			Body size: locomotion	11.39 (6)	0.07	

Abbreviation: GLM, generalised linear model.

^a^ All main effects retained for all models.

^b^ Three‐way interactions were included, but subsequently removed from all models during the backwards selection process.

^c^ Residual deviance and *df* for final model.

^d^ Diameter and orientation refer to support diameter and support orientation.

When moving arboreally, the gorillas used small supports (<10 cm) more than one third of the time (Table 9). Large supports (>20 cm) were the second most frequently used support size, accounting for 28%, and medium supports (10–20 cm) were used 21% of the time. When walking, the gorillas used all support sizes in frequencies as expected, with a slight preference for small supports over larger supports. When engaging in vertical climbing, large supports were used much more than expected (SCR = 4), but small supports were used much less than predicted (SCR = −3.5). The gorillas used small supports more than 60% of the time when engaging in suspension and gap‐crossing, which was much higher than expected (SCR = 4.6 and 4.1). Conversely, for both suspension and gap‐crossing, large supports were used <5% of the time, much less than expected (SCR = −4.7 and −3.8).

**TABLE 9:** Contingency table for the interaction between locomotion and support diameter (for description, see Table 7).

Locomotion	Support diameter				
	Small	Medium	Large	Mixed	Total
Walking	34.5 (38.2)	20.7 (38.9)	27.9 (39.8)	16.9 (45.2)	39.8
Vertical climbing	26.1 (31.2)	24.8 (50.3)	37.9 (58.3)	11.3 (32.6)	43.0
Suspension	65.6 (19.3)	15.6 (7.8)	2.1 (0.8)	16.7 (11.9)	10.6
Gap‐crossing	61.7 (11.3)	10.0 (3.1)	5.0 (1.2)	23.3 (10.4)	6.6
Total	36.0	21.2	27.9	14.9	100

*Note*: 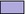, SCR > +2; 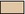 SCR < −2.

Abbreviation: SCR, standardised cell residuals.

**FIGURE 7:**
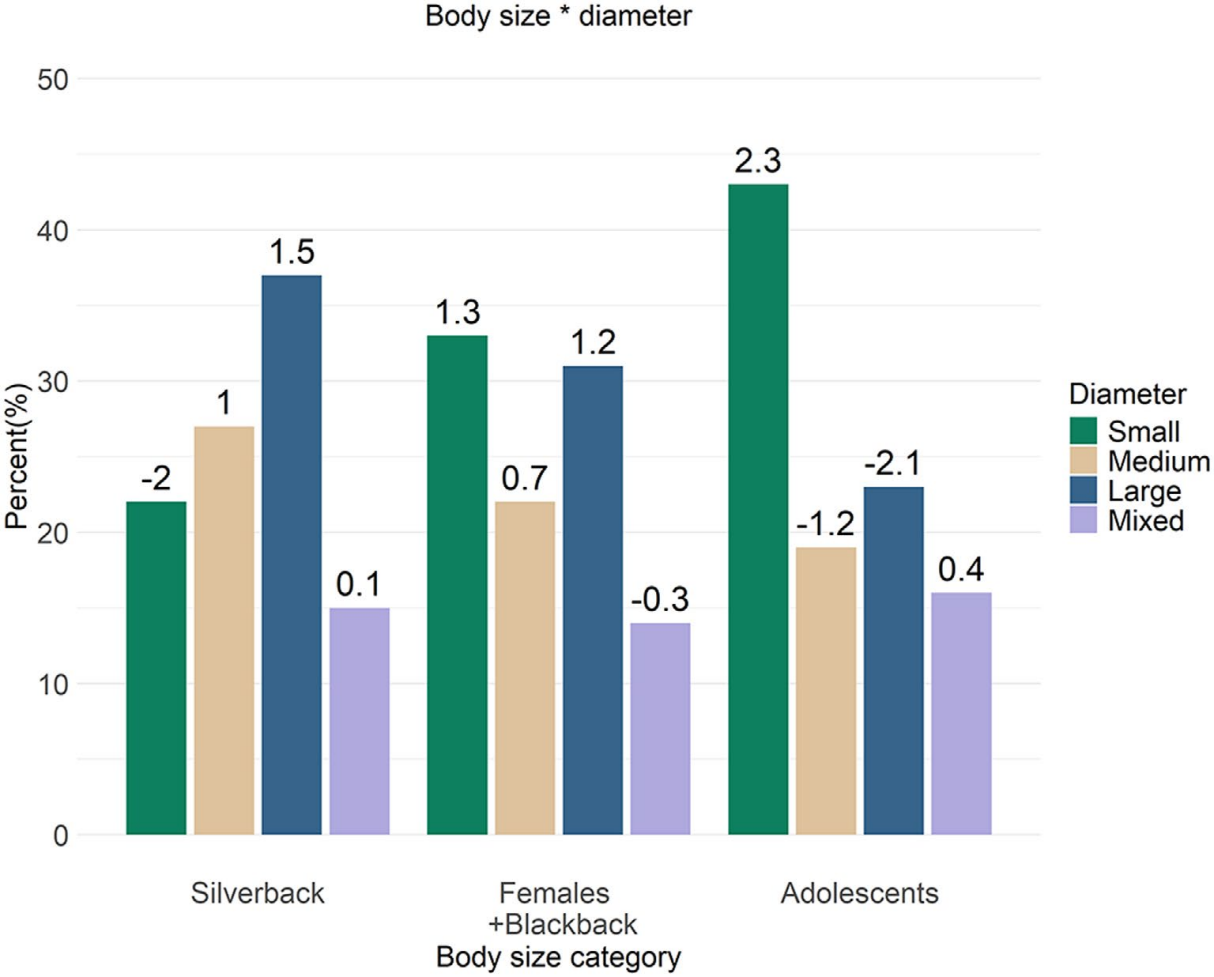
Model interaction between body size category and support diameter. The values above the bars represent the standardized cell residuals (SCRs).

Body size was significantly associated with the support diameter variable, suggesting that the variation in size between the silverback, females and adolescents is linked to differences in the size of supports used, but not orientation, number of supports and hand position. Figure 7 illustrates the percentiles and SCRs for the interaction between body size and support diameter. For larger gorillas, locomotion on small supports decreased and locomotion on large supports increased. The silverback, females and blackback used supports of all sizes in frequencies as expected. Conversely, the adolescents used small supports more than expected (SCR = 2.3), accounting for more than 40% of all supports, but used large supports less than expected (SCR = ‐2.1).

The gorillas used tree branches 78% of the time when moving arboreally, 3.5 times more than any other support type. Branch bundles were the second most frequently used support type, accounting for 9%, followed by lianas, which were used 7% of the time (Table 10). Tree trunks were the least frequent support type, accounting for only 1.6% of all supports. When walking, the gorillas used branch bundles more than expected (SCR = 2.1), but lianas much less than expected (SCR = −4.2). Conversely, when engaging in vertical climbing, branch bundles were only used 4.5% of the time, much less than predicted (SCR = −4.6), but lianas were used more (SCR = 5.6). When engaging in suspension, the gorillas used all support types within the expected range, with the exception of liana/branch combinations, which accounted for 12% of all suspension (SCR = 4.5). The gorillas crossed gaps on all support types except for tree trunks, but all support types fell within the expected frequencies for this locomotor behaviour.

**TABLE 10:** Contingency table for the interaction between locomotion and support type (for description, see Table 7).

Locomotion	Support type					
	Liana	Liana/branch	Branch	Trunk	Bundles	Total
Walking	2.7 (15.8)	3.2 (32.7)	80.9 (45.3)	1.6 (42.9)	11.7 (55.9)	43.5
Vertical climbing	14.3 (70.5)	3.0 (25.5)	75.7 (35.4)	2.6 (57.1)	4.5 (17.8)	36.3
Suspension	4.0 (7.4)	12.1 (38.2)	71.1 (12.2)	0	12.7 (18.6)	13.3
Gap‐crossing	6.7 (6.3)	2.2 (3.6)	80.9 (7.1)	0	10.1 (7.6)	6.9
Total	7.3	4.2	77.7	1.6	9.1	100

*Note*: 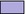, SCR > +2; 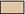, SCR < −2.

Abbreviation: SCR, standardised cell residuals.

Table 11 shows the percentages and SCRs for the interaction between locomotion and support orientation. When walking, the gorillas predominantly used horizontal supports, in frequencies higher than expected (SCR = 10), but vertical supports much less than expected (SCR = 12). They also used multiple supports of mixed orientations more than expected (SCR = 2.9). Vertical climbing was not restricted to vertical supports, but they were used considerably more than all other orientations (SCR = 13.6). Unlike walking and vertical climbing, suspensory locomotion and gap‐crossing saw a more varied use of different support orientations. The gorillas used suspension to travel in approximately equal amounts on horizontal, angled and vertical supports, but U‐shaped lianas (U‐shape supports were exclusively lianas) were used much more than expected (SCR = 4.6), along with angled supports (SCR = 3.7). When gap‐crossing, the gorillas used mostly vertical supports, but all supports were used in frequencies as predicted.

**TABLE 11:** Contingency table for interaction between locomotion and support orientation (for description, see Table 7).

Locomotion	Support orientation					
	Horizontal	Angled	Vertical	U‐shaped	Mixed	Total
Walking	55.8 (78.9)	16.1 (54.3)	1.4 (1.6)	1.9 (32.0)	24.8 (52.7)	41.3
Vertical climbing	4.7 (6.3)	5.2 (16.5)	77.6 (81.6)	0.7 (12.0)	11.7 (23.4)	38.7
Suspension	23.4 (10.9)	21.3 (23.6)	26.2 (9.7)	9.2 (52.0)	19.9 (13.9)	13.6
Gap‐crossing	17.9 (4.0)	10.4 (5.5)	40.3 (7.1)	1.5 (4.0)	29.9 (10.0)	6.5
Total	29.2	12.2	36.7	2.4	19.4	100

*Note*: 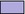, SCR > +2; 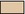, SCR < −2.

Abbreviation: SCR, standardised cell residuals.

### 3.4 Number of supports

Model 3 tested whether the gorillas of different body size groups used more supports when engaging in different locomotor behaviours or at different heights (Table 8). Body size was not associated with the number of supports, but instead, the gorillas used different amounts of supports when moving in different locomotor behaviours and at different heights. Overall, the gorillas used a single support more than multiple supports, accounting for 66% of their arboreal locomotion (Table 12). Single supports were used more than predicted during vertical climbing (SCR = 3.1), but less than predicted during gap‐crossing (SCR = −2.5). The gorillas walked and gap‐crossed on 2–4 supports more than predicted (SCR = 2.3 and 2.6). More than four supports were used more than predicted during suspensory locomotion and gap‐crossing (SCR = 3.5 and 2.4).

**TABLE 12:** Contingency table for the interactions between number of supports with locomotion and height (for description, see Table 7).

	No. of supports			
	1	2–4	>4	Total
Locomotion				
Walking	58.2 (36.4)	31.3 (50.8)	10.4 (50.0)	41.2
Vertical climbing	78.6 (49.0)	18.7 (30.2)	2.7 (13.1)	41.1
Suspension	62.1 (11.2)	19.8 (9.3)	18.1 (25.0)	11.9
Gap‐crossing	39.3 (3.4)	42.9 (9.7)	17.9 (11.9)	5.7
Total	65.9	25.4	8.6	100
Height (m)				
<10	67.9 (63.1)	26.0 (63.9)	6.0 (45.6)	61.9
10–20	63.2 (29.8)	24.3 (30.3)	12.5 (48.1)	31.4
>20	70.8 (7.1)	21.5 (5.7)	7.7 (6.3)	6.7
Total	66.6	25.2	8.2	100

*Note*: 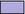, SCR > +2; 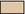, SCR < −2.

Abbreviation: SCR, standardised cell residuals.

At lower heights, the gorillas used >4 supports less than predicted by the model (SCR = 2.0), they used this amount of supports more than predicted at mid‐heights (SCR = 2.6). The interaction between number of supports, locomotion and height was removed from the modelling process, which suggests that height did not influence the number of supports used for each locomotor behaviour.

### 3.5 Hand posture and grip

Model 4 tested whether the gorillas altered their hand posture for specific locomotor behaviours and whether hand posture varied for different body size groups (Table 13). Hand posture was not associated with body size, but instead was associated with locomotion and support diameter. During tensile locomotor behaviours, on small supports, the gorillas used a diagonal power grip considerably more than expected (SCR = 7.8), but power grips much less (SCR = −11.2). Similarly, on branch bundles, the gorillas used diagonal power grips more than predicted (SCR = 4.5). When using medium and large supports, power grips were used much more than expected (SCR = 2.5 and 13.2).

**TABLE 13:** Contingency table for the interaction between support diameter and hand posture split by tensile and compressive locomotion (for description, see Table 7).

Hand posture	Support diameter				
	Small	Medium	Large	Bundles	Total
Tensile					
Diagonal power grip	64.3 (93.8)	18.5 (55.4)	0.6 (1.4)	16.6 (97.2)	60.6
Diagonal finger grip	86.7 (3.0)	0	0	13.3 (1.8)	1.4
Power grip	3.5 (3.2)	23.7 (44.6)	72.6 (98.6)	0.2 (0.9)	38.0
Total	41.5	20.2	28.0	10.3	100
Compressive					
Knuckle	21.4 (18.4)	33.3 (23.3)	44.0 (40.7)	1.2 (4.2)	25.2
Fist	15.6 (5.1)	34.4 (9.2)	46.9 (16.5)	3.1 (4.2)	9.6
Palm	34.6 (76.5)	37.3 (67.5)	18.0 (42.9)	10.1 (91.7)	65.2
Total	29.4	36.0	27.3	7.2	100

*Note*: 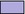, SCR > +2; 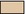, SCR < −2.

Abbreviation: SCR, standardised cell residuals.

When the gorillas engaged in compressive, above‐branch locomotion, they contacted supports using knuckle‐down, fist‐down and palm‐down postures. As support diameter increased, the frequency of knuckle and fist use increased. On small supports, the gorillas used knuckles less than expected (SCR = −2.5), but palms much more (SCR = 2.4). Hand posture deviated the least on medium supports and exhibited the least amount of variation. On large supports, the gorillas used palms less than expected (SCR = −4.2) but knuckles more than expected (SCR = 5), accounting for more than 60% of locomotion on large supports. When moving on branch bundles, the gorillas used knuckle postures much less (SCR = −2.5), but palm contact much more than predicted (SCR = 2.1).

## 4 DISCUSSION

Gorillas are the largest ape that exploits arboreal environments to access nutritional resources. However, they also possess postcranial adaptations towards terrestrial quadrupedalism, which have been shaped by natural selection in response to the biomechanical demands of travelling on the ground (Buschang, 1982; Gebo, 1996; Gregory & Raven, 1950; Roberts, 1974; Tuttle, 1967). It was not previously understood how, given their size and morphology, gorillas address the ecological challenges of accessing, moving around and dealing with discontinuity in the canopy. In our study, the western lowland gorillas in Loango spent 11% of their arboreal time engaging in locomotion to achieve the goal of feeding on arboreal resources. The results of our analyses suggest that the gorillas' arboreal repertoire is influenced by a complex interplay between locomotor behaviours, support variation, hand posture and height in the canopy. We also present evidence that indicates that body size does play a role in shaping the arboreal repertoire of western lowland gorillas, since body size was associated with variation in locomotor behaviour, height and the size of supports used. Tree canopies are characterised by unpredictability, which is accompanied by significant risk, but gorillas of all body sizes were able to adapt their arboreal behaviours to solve the problems of acquiring nutritional resources. We contextualise our findings using the ecomorphology framework in order to better understand the role of environment, morphological adaptations, risk and energetic expenditure in shaping the arboreal behaviour of this species.

### 4.1 Body size and arboreality

The pursuit of arboreal resources is one of the main motivations for western lowland gorillas to venture from less challenging terrestrial environments into the trees (Robbins et al., 2022). Locomotion during foraging in the trees is one of the most challenging activities in which primates engage because it normally takes place in the periphery of tree canopies, which are typified by supports that are discontinuous, unstable and small (Schmidt, 2011). Given their large body size, gorillas should be extremely cautious when foraging and feeding in these environments. It is often theorised that large primates should switch from above‐branch locomotion to suspensory locomotion when branches become small because they are less likely to rotate off the support (Cartmill & Milton, 1977). Suspensory locomotion is associated with travelling on small supports in orangutans (Thorpe & Crompton, 2005), so it was expected that the gorillas may also resort to suspension when accessing arboreal resources. However, the gorillas instead switched from quadrupedal walking to bipedal walking. Bergeson (1998) suggested that an alternative way for arboreal primates to solve problems of balance on small branches is to use multiple supports to balance above rather than below the primary weight‐bearing supports. We found this is the primary strategy utilised by the largest arboreal ape. The gorillas were able to engage in foraging and feeding on small supports using hand‐assisted flexed‐hindlimb bipedal walking, as they could free‐up one hand while grasping multiple supports with the other limbs. Hindlimb compliance (flexed limbs) allows them to lower their centre of mass and reduce vertical oscillations of small supports, increasing stability (Schmitt, 1999). The compliant gait of gorillas and chimpanzees in arboreal environments is considered to be advantageous to their success in environments in which they are not fully specialised (Larney & Larson, 2004; Schmitt, 1999; Tarrega‐Saunders et al., 2021). However, while hand‐assisted bipedal walking increases their safety when feeding as it reduces the risk of falling if a branch snaps, it may have come at the cost of increased energy expenditure. Comparative energy expenditure during different locomotor behaviours, especially in arboreal environments, is not fully understood despite its relevance for primate ecomorphology (Arnold, 1983; Bock, 1994; Bock & von Wahlert, 1985; Wainwright, 1991). However, bipedal walking in a bent‐hip, bent‐knee position is likely accompanied by increased energetic costs because of the requirement for constant muscular effort (Carey & Crompton, 2005; Crompton et al., 1998; Kozma et al., 2018; Pontzer et al., 2009; Sockol et al., 2007). Suspensory locomotion, on the other hand, is perhaps less metabolically expensive because although it requires muscular engagement in the forelimb and hand to grasp the support, gravity helps keep the body in position. This suggests that gorillas might experience a trade‐off between safety and energy efficiency when acquiring arboreal resources. This compromise may be less pronounced for smaller primates as their size allows them easier access to the terminal branch niche and moving on small supports.

In addition to being the largest arboreal primate, gorillas also exhibit the highest degree of sexual dimorphism, with silverback gorillas being much larger than adult females. This has an evolutionary advantage, as larger silverbacks are more successful in competing for mates and protecting their group from predators and during intergroup conflict (Breuer et al., 2016; Cassini, 2020). The females' smaller size allows them to be more agile and reduce their overall calorific requirements. We observed, similarly to Remis (1995), that the silverback would often feed on the ground rather than venturing into the trees with the females and adolescents. The energetic cost of locomotion is thought to be directly linked to the amount of force produced by the muscles to move the body (Alexander, 1985; Fenn, 1938). Larger primates have less muscle force per unit mass (Fleagle, 1985), so they experience higher absolute costs during locomotion than smaller primates. Vertical climbing, in particular, is more energetically costly for large primates because of the mechanical work and muscular effort involved in opposing gravity relative to their size (Fleagle, 1985; Hanna et al., 2017; Hanna & Schmitt, 2011; Isler, 2005; Pontzer & Wrangham, 2004). Although the silverback gorilla engaged in proportionally more vertical climbing than other individuals (perhaps due to constraints in his ability to move horizontally as supports taper and become compliant), he may have spent more time on the ground because of the increased energetic costs involved in climbing. However, this could also be influenced by his social role, as silverbacks play a protective role within the group, remaining on the ground while the females and adolescents feed arboreally. The silverback also spent only 2% of his time at heights above 20 m; four times less than the females and six times less than the adolescents. In general, smaller primates face fewer limitations in terms of substrate size or forest layer, especially in the emergent canopy, where nutritional resources are often located (McGraw, 1998). The adolescent gorillas were likely able to choose between a more diverse array of canopy use patterns such as heights above 20 m because of their smaller size.

When in the trees, the silverback spent only 7% of his time engaging in locomotion, compared to 11% for the females and 13% for the adolescents. In complex arboreal environments, the silverback might be more constrained in his ability to move around because the branches and foliage create dense, intertwined networks. This may in turn increase the risk of falling. Falling has more serious consequences for larger individuals, especially at higher heights, as they have a higher chance of fatality because the kinetic energy that the body has to dissipate upon impact increases (Cartmill & Milton, 1977; Preuschoft et al., 2016). We therefore predicted that there would be variation in the supports used by gorillas of different body sizes to mitigate the risk of falling, similar to many other primates, including Surinam monkeys (Fleagle & Mittermeier, 1980), tamarin monkeys (Garber, 1991), lemurs (*Lepilemur edwardsi* and *Avahi occidentalis*) (Warren & Crompton, 1997), sympatric Old World monkeys in the Tai forest (McGraw, 1998) and Western tarsiers (Crompton, Blanchard, et al., 2010). The results instead highlight that gorillas of all body sizes used a similar type, orientation and number of supports when moving arboreally. However, in order to reveal whether gorillas of all body sizes used similar supports because of a preference towards them, or whether use reflects the supports that were available, more data is required on support availability. Our understanding of orangutan locomotion in Borneo and Sumatra has benefitted significantly from data on support availability (Manduell et al., 2012).

We did find, however, that the size of supports used was associated with a variation in body size. For orangutans and chimpanzees, support size did not significantly vary between body size or age–sex classes (Hunt, 1992a, 1992b; Thorpe & Crompton, 2005). Thorpe and Crompton (2005) suggest that for orangutans, this reflects the presence of arboreal pathways which are used by all orangutans when travelling to large fruiting trees. However, unlike the other great apes, adult male gorillas weigh around 100 kg more than their female counterparts, so it is not surprising that the silverback used large supports more than the females and blackback. In contrast, the adolescents used small supports twice as often as the silverback and 1.2 times more than the adult females and blackback. These results might seem to support the classic prediction that larger primates will tend to use larger supports than smaller primates (Cant, 1992; Cartmill & Milton, 1977). However, a support is only ‘large’ or ‘stable’ relative to a primate's body size (Avis, 1962), so this variation in size means that the gorillas effectively used supports that were large‐enough and stable‐enough to bear their weight.

### 4.2 Age–sex variation in arboreal risk sensitivity

Crossing gaps in the canopy is an ecological challenge that must be addressed for a species to be successful when exploiting arboreal environments (Halsey et al., 2016; Pontzer & Wrangham, 2004). Rather than descending to the ground to travel to nearby feeding sites (as documented by Remis, 1995), the gorillas in this study crossed gaps 7% of the time when moving in the trees. However, contrary to other locomotor behaviours, larger gorillas did not use larger supports to cross gaps, presumably because these supports are often not available where gaps are located in the canopy (Druelle et al., 2020; Graham & Socha, 2020). Instead, all of the gorillas crossed gaps in the trees on small supports but mitigated the risk of falling by using multiple small supports of different orientations. While younger apes tend to show a greater degree of acrobatic dynamic locomotion than mature individuals (Doran, 1997; Thorpe & Crompton, 2005), the risks involved in gap‐crossing on small supports might explain why the smallest age–sex class, the adolescents, engaged in gap crossing the most, accounting for 9% of their overall arboreal repertoire. Conversely, the females, blackback and silverback crossed gaps 6% of the time. However, this does not imply that larger gorillas are more cautious: instead, when looking at *how* the gorillas crossed gaps, it is likely that the females were more cautious in their gap‐crossing. While the silverback was not observed to engage in bridging, he crossed gaps by leaping (40%) and riding branches (60%). In contrast, the females and blackback bridged gaps 55% of the time, twice as often as oscillating branches and rarely crossed gaps by leaping. The adolescents were the only body size group which crossed gaps above heights of 20 m, and overall engaged in bridging most frequently (45%) followed by leaping (35%) and branch riding (20%). Bridging a gap is the most secure way to cross a gap (that cannot be crossed using suspension) because it allows a primate to slowly transfer onto the supports on the other side of the gap while testing their capacity to bear their weight. This makes the transition to the new supports much less risky than in leaping or ride and sway (Fleagle & Mittermeier, 1980). All the females in this study were carrying infants under the age of two. Therefore, it is possible that their lower propensity to engage in gap‐crossing, their tendency to cross gaps at lower heights, and their preference for more secure ways of navigating tree discontinuities were influenced by the need to prioritize safety while carrying infants. Adult female orangutans have also been observed to be more cautious when moving in the trees than adult males and adolescents (Thorpe & Crompton, 2005), suggesting that the reproductive status of female apes may influence the way in which they view and attend to risks. In addition, infant‐carrying is metabolically more expensive, especially as the weight of the infant increases, which might also impact their choice of routes while moving in unstable environments (Anvari et al., 2014; Goto et al., 2022; Schradin & Anzenberger, 2001). The females may have opted to cross gaps using the safest strategy of bridging, even though it is more energetically costly compared to ‘ride’ and ‘sway’ (Thorpe, Crompton, & Alexander, 2007).

Crossing gaps in arboreal environments using branch oscillation accounted for 28% of the gorillas' gap‐crossing behaviours. The oscillation of branches during ‘ride’ and ‘sway’ is a strategy used by orangutans to cross gaps by using their momentum to sway compliant branches to minimise path lengths and optimise their daily energetic cost of locomotion (Manduell et al., 2011; Thorpe et al., 2009; Thorpe, Crompton, & Alexander, 2007; van Casteren et al., 2013). Although manipulating supports using branch ride and sway requires advanced cognitive abilities and experience (Chevalier‐Skolnikoff et al., 1982), for orangutans the risks of branches breaking seem to be outweighed by the reduced energetic cost compared to other gap‐crossing behaviours (Thorpe, Crompton, & Alexander, 2007). We present new evidence to show that gorillas also engage in branch oscillation in the wild. Branch ‘riding’ accounted for 88% of their oscillatory locomotion, with ‘sway’ representing only 12%. However, the relationship between risk and energetic expenditure is difficult to determine. Interestingly, the gorillas crossed arboreal gaps using oscillation exclusively at heights <20 m (the silverback used heights of <10 m). While orangutans spend more time at heights above 20 m (35% of their arboreal repertoire compared to 9% for gorillas), 90% of their oscillatory locomotion occurred at heights below 20 m (Thorpe & Crompton, 2005). Gorillas might be more cautious in their approach to oscillatory gap‐crossing on compliant supports, but there might also be interspecific similarities in oscillation at different heights. In order to determine whether this reflects cautiousness or habitat structure, we need to integrate more data on the supports that are available in Loango.

Risk sensitivity might also influence the gap‐crossing strategies used by adolescent gorillas. The adolescent gorillas were the only age–sex group that crossed gaps at the highest layer in the canopy. The exploratory stage of infant and adolescent primates is a key stage in their developmental process as it requires them to take risks in order to develop their balance, coordination and strength (Doran, 1997; Dunbar & Badam, 1998; Hurov, 1991; Steenbeek et al., 1996). Infant gorillas will often climb on small trees when their mothers are on the ground, and it is common for them to frequently fall off during these early attempts (Doran, 1997 for mountain gorillas; Martha Robbins, pers. comm., 2024 for western lowland gorillas). Adolescents are not dependent on their mothers, but their development is shaped by independent exploration and play behaviours with other adolescents in the trees (locomotion during playing accounted for 4% of the adolescents' arboreal repertoire). Adolescent gorillas are also more agile and experience less risk of severe injury from falling than adults (Cartmill & Milton, 1977; Preuschoft et al., 2016). This might be why, when gap‐crossing, the adolescents engaged in leaping 35% of the time. Leaping is the most risky gap‐crossing behaviour because the support that is leaped onto must be strong enough to withstand the impact of landing. It is also highly energetically costly if the take‐off support is compliant, as they lose energy to the support during push off (Crompton et al., 1993; Demes et al., 1995, 1999). Small‐bodied leaping primates likely possess adaptations for leaping so they can quickly escape predators in discontinuous forests (Cartmill, 1974; Fleagle, 1985). Although the silverback was observed to leap when fleeing from supports that were collapsing under his weight, the adolescents leapt during social play behaviours, to avoid wasps' nests, and to move onto neighboring trees when being chased by the more dominant females and silverback, even at heights of more than 20 m. Thus, although all gorillas have the mechanisms to leap, there were age–sex differences that likely reflect the risks involved in leaping in the trees.

### 4.3 Gorilla hands: A compromise morphology?

The hand morphology of gorillas has received much attention because it is often regarded as the most specialised aspect of their anatomy, facilitating quadrupedal walking on the ground (in this study, accounting for 95% of their terrestrial locomotor repertoire) (Jenkins & Fleagle, 1975; Matarazzo, 2013; Tuttle, 1967; Zihlman et al., 2011). Gorillas possess the highest thumb‐forefinger index of the non‐human great apes (Schultz, 1930). The fact that they resemble hominins in this respect as well as in pedal digital proportions (Schultz, 1930) suggests that this is the ancestral African great ape condition. Nevertheless, it also increases stability and control in quadrupedal walking and helps to dissipate compressive forces (Gebo, 1992; Schultz, 1963; Tuttle, 1967). Similar manual digital proportions allow gorillas as well as humans to oppose their thumbs, but as this is most unlikely to enhance efficiency during quadrupedal terrestrial walking, it is probably an adaptation for hand‐supported orthograde arboreality. However, to what extent, if any, their hand dimensions might constrain their ability to move in arboreal environments will depend on the behaviours engaged in.

Although the biomechanics of hand postures during quadrupedal walking and vertical climbing have received much attention from a morphological and evolutionary perspective (Kivell & Schmitt, 2009; Neufuss et al., 2017; Tarrega‐Saunders et al., 2021), there has to date been a notable gap in our understanding of the relationship between hand morphology and arboreal locomotion of wild western lowland gorillas. The results in the present study revealed that there was no relationship between body size and hand posture when moving in arboreal environments, despite silverback males being much larger in size. Hand posture was, however, associated with locomotor behaviour and support size. When walking quadrupedally, hand posture was slightly variable, but the general pattern was that knuckle and fist postures were used more on large supports (>20 cm in diameter), perhaps because these supports are similar to terrestrial environments, where knuckle and fist postures dominate (Thompson et al., 2018). However, when walking on small supports (<10 cm in diameter), the gorillas used palmar hand postures. These patterns have also been observed for other primates, including chimpanzees during arboreal quadrupedal walking (Hunt, 1991; Preuschoft, 2002). Using a palmar grip on small branches allows the thumb to grip onto the other side of the support, so if it begins to oscillate or rotate, the gorillas can create ‘frictional resistance’ to avoid toppling to the side (Preuschoft, 2002). Palmigrady also reduces the stress on individual hand bones, as it distributes weight and pressure across the volar pads evenly (Patel & Wunderlich, 2010). There was also a strong association between hand postures and support size during vertical climbing. When engaging in vertical climbing on large supports, the gorillas used power grips (no thumb opposition), whereas on small supports, they used diagonal power grips, where the thumb would also be engaged to help ascend or descend supports, in a similar manner to observations for mountain gorillas and chimpanzees (Neufuss et al., 2017). The hand dimensions of gorillas allow them to climb both small supports, using thumb opposition, *and* large supports using full hand contact, which may be more difficult for orangutans because of their increased manual curvature. The gorillas in this study mostly used large supports (38%) when engaging in vertical climbing, but they also used small and medium sized supports relatively often (25%–26%). This suggests that their high thumb‐forefinger index permits them to adopt a flexible approach when accessing different heights in the trees.

Over the course of evolutionary history, gorillas have not lost complete mobility in the wrist joint. Likewise, they have not lost all carpal and phalangeal curvature or the large flexor sheath ridges which allow for more robust flexor muscles to assist in gripping supports in tensile positions (as in orangutans) (Sarmiento, 1988; Susman, 1979). However, despite possessing these traits towards strong grasping strength for behaviours such as suspension, we found that their hand dimensions restricted the gorillas to suspension on small supports (66% of supports used during suspension). In fact, the gorillas used small supports twice as often as orangutans when engaging in suspensory locomotion (Thorpe & Crompton, 2005). Typically, they used multiple small supports of different orientations, branch bundles (clusters of intertwined branches) and small branches in combination with lianas, presumably to mitigate for their size. Western lowland gorillas have rarely been observed to use lianas (Remis (1995) documented that the gorillas used lianas only 1% of the time), but in this study, lianas in combination with tree branches were used much more than predicted during suspension, accounting for 16% of their suspensory locomotion, which was more than any other arboreal locomotor behaviour. Furthermore, branch bundles, used frequently by orangutans, also represented 9% of all arboreal support types used by the gorillas. Studies of orangutans (Thorpe & Crompton, 2005) have shown that holding clusters of branches enhances stability and reduces the risk of falling due to support‐breakage. This suggests that the gorillas were able to compensate for their hand proportions and size by adopting a flexible approach to moving horizontally through the canopy on a variety of multiple supports.

### 4.4 Implications for hominoid bipedal evolution

The evolution of hominoid bipedal locomotion has received much attention and has been heavily debated within palaeoanthropological literature for over a century. Several models have been put forward which offer different perspectives on how early hominoids may have transitioned to bipedal walking (for a comprehensive review of these models, see for example, Crompton et al., 2008). All apes are capable of (and will occasionally engage in bouts of) bipedal walking, but they also exhibit highly variable locomotor repertoires and anatomy (Doran, 1996; Hunt, 1991; Thorpe et al., 2014; Thorpe & Crompton, 2005; Tuttle & Watts, 1985). We can use extant nonhuman apes as referential models for understanding the relationship between locomotor performance and postcranial morphology in wild environments (Almecija et al., 2021; Crompton, 2016; Crompton, Sellers, & Thorpe, 2010; Hunt, 1994; Kozma et al., 2018; Pilbeam & Lieberman, 2017; Richmond et al., 2001). This can then be applied to what we know about the fossil record and allow us to develop our understanding of the bipedal body plan. Gorillas, in particular, are suitable models because not only are they similar in their hand (and foot) proportions to modern humans (and indeed early hominins), but they also habitually use arboreal and terrestrial environments (Remis, 1995). Learning how they can adapt to varying environments may offer a new perspective on the anatomical and behavioural shift that occurred during hominoid evolution.

Orthograde postures play an essential role for chimpanzees and gorillas when feeding in trees (Hunt, 1994) and enable orangutans to access terminal branch niches where supports are thin and flexible (Crompton, Sellers, & Thorpe, 2010; Thorpe, Crompton, & Alexander, 2007). Orthograde (torso‐vertical) postures have been documented to dominate the locomotor repertoire of orangutans (Thorpe & Crompton, 2005), which is consistent with their postcranial musculoskeletal adaptations towards orthograde suspensory postures and locomotion. In this study, we found that the gorillas in Loango adopted torso‐orthograde locomotion 46% of the time when in the trees, despite the high frequency of torso‐pronograde locomotion when on the ground. The use of torso‐orthograde postures by gorillas in arboreal environments demonstrates significant behavioural plasticity. Unlike orangutans, gorillas rely on behavioural flexibility to overcome challenges posed by their less arboreally specialized morphology, providing them nevertheless with an ability to adapt to varying ecological pressures such as navigating discontinuous canopies and accessing resources on compliant supports. This capacity for behavioural adaptation highlights the importance of plasticity in achieving ecological and evolutionary success (West‐Eberhard, 2005). Behavioural flexibility allows gorillas to compensate for morphological traits that are not optimized for arboreal environments, solving the unique challenges of arboreal locomotion through behavioural strategies rather than specialized anatomical adaptations.

Despite gorillas being the original model species for the knuckle‐walking hypothesis (Washburn, 1967), the behavioural flexibility documented in our study supports an arboreal orthograde model (Crompton et al., 2008; Crompton, Sellers, & Thorpe, 2010; Thorpe, Holder, & Crompton, 2007) rather than knuckle‐walking as the likely precursor to bipedalism. Indeed, recent biomechanical analysis of quadrupedal walking has also shown that while the African apes do exhibit some general similarities in their knuckle‐walking, there are significant interspecific kinematic distinctions (Inouye & Shea, 2004; Kivell & Schmitt, 2009; Tarrega‐Saunders et al., 2021). This implies that knuckle‐walking is either a shared behavioural flexibility or evidence of independent evolution as a response to environmental pressures, but likely not the locomotor condition which transitioned into bipedalism (Tarrega‐Saunders et al., 2021). We already know that the great apes differ in their biomechanics when using orthograde locomotion, as orangutans use extended‐hindlimb bipedalism (Crompton, Sellers, & Thorpe, 2010; Thorpe & Crompton, 2005) whereas this study documents gorillas as engaging in flexed‐hindlimb bipedalism. This suggests that although great apes likely shared evolutionary pressure to use orthograde postures in the trees, they may have developed distinct biomechanics within these behaviours because of local environmental pressures. This led to the adoption of vertical climbing in gorillas and chimpanzees as a route to partial terrestriality in deep tropical forest while hominins exploited forest margins to a greater extent. Overall, however, our new understanding of how gorillas move in the trees is consistent with the idea that bipedalism on the ground may indeed have transitioned from orthograde postures in the trees (Crompton, Sellers, & Thorpe, 2010; Thorpe et al., 2014; Thorpe, Holder, & Crompton, 2007).

## 5 CONCLUSION

We have provided new evidence to show that the western lowland gorillas are not restricted in their access to, or manoeuvrability within tree canopies, despite their large size. Rather, we found that the locomotor behaviours used by gorillas of different body sizes contradicted classic body size predictions for arboreal primates (Cartmill & Milton, 1977). The gorillas did not rely on suspensory locomotion to move horizontally on small supports in the canopy as much as predicted; instead, they frequently opted for hand‐assisted bipedal walking. This is perhaps because suspensory locomotion on small supports is challenging for gorillas (even the silverback) because of their hand dimensions and size. While larger gorillas tended to use larger supports overall, engaged in less horizontal locomotion, and spent less time locomoting in the emergent canopy compared to smaller gorillas, gorillas of all body sizes used a similar number and type of supports. However, age–sex classes may also have played a role in how risks were attended to. Infant‐carrying females used the most secure gap‐crossing behaviours despite them being metabolically demanding. Overall, we conclude that the gorillas likely prioritised risk minimisation over energetic expenditure.

This new evidence suggests that western lowland gorillas were not limited in their arboreal locomotion and use of orthograde postures by a postcranial morphology that is highly effective in terrestrial quadrupedal walking. Instead, they exhibited considerable behavioural flexibility in order to feed in arboreal environments. However, morphology and behaviour are much easier to quantify than ecology as it is often difficult to define the environment of a species because a single ecosystem can include many habitat types (Elton et al., 2016). To further contextualise our findings and fully assess the relationship between functional anatomy and the environment, we need to obtain more data on support availability and integrate this into future research. From an evolutionary perspective, this can help us better understand the role of the environment and the selective pressures that led to the transition in locomotor behaviours that we see in extant great apes today.

## AUTHOR CONTRIBUTIONS

Conceptualisation: Charlotte A. King and Susannah K. S. Thorpe. Subject information: Martha M. Robbins. Data collection: Charlotte A. King. Statistical analysis and interpretation of results: Charlotte A. King, Susannah K. S. Thorpe and Jackie Chappell. Manuscript – original draft: Charlotte A. King. Manuscript – review and editing: Susannah K. S. Thorpe, Martha M. Robbins, Robin H. Crompton, Jackie Chappell, and William I. Sellers. Illustrations: Charlotte A. King.

## Supporting information


Appendix S1

## Data Availability

The data that support the findings of this study are available from the corresponding author upon reasonable request.
