# Supplementary material for: The locomotor ecology of wild western lowland gorillas: How does the largest ape exploit complex arboreal environments?
Source: J Anat. 2025 May 9;248(2):197–221. doi: 10.1111/joa.14277 (PMC12779408; doi:10.1111/joa.14277)
Supplement: Supplementary file 1 — Appendix S1 [file JOA-248-197-s001.docx]

# SUPPLEMENTARY MATERIAL

Descriptions of locomotor submodes described by Hunt et al., (1996) and developed by Thorpe and Crompton (2005) with percentage for each submode (for all gorillas arboreally).

| Locomotor mode, *submode,* description | % |
| --- | --- |
| Quadrupedal walk |  |
| *Symmetrical gait walk,* regular (usually diagonal sequence, diagonal couplets) gait sequence | 14.05 |
| *Irregular gait walk,* scramble, lacking a regular gait | 13.97 |
| *Quadrupedal run irregular,* fast, where footfall patterns follow no regular sequence | 0.07 |
| Tripedal walk |  |
| *Tripedal walk,* same as quadrupedal, except one limb is not used | 0.67 |
| Bipedal walk |  |
| *Extended bipedal walk,* hip and knee are extended | 0.29 |
| *Flexed bipedal walk,* hip and knee are relatively flexed | 0.67 |
| *Hand assisted extended bipedal walk*,* hindlimbs bear more than 50% of body mass in full extension, but one or both forelimbs as used to assist, either in suspension or compression and bear more than their own weight | 5.0 |
| *Hand assisted flexed bipedal walk*,* as for ‘hand assisted extended bipedal walk’, but hindlimbs are relatively flexed | 5.74 |
| *Bipedal scramble*,* hindlimbs bear majority of body mass but hindlimb kinematics are not characterised by a regular sequence | 0.30 |
| *Hand assisted bipedal scramble*,* as for ‘bipedal scramble’, but with hand assistance | 3.36 |
| Vertical climb |  |
| *Flexed-elbow vertical climb,* propulsion provided by hindlimbs as well as forelimb flexion to pull body upwards | 8.52 |
| *Ladder climb,* as for ‘flexed-elbow vertical climb’, but except supports are often relatively horizontal and never a single vertical support | 0.37 |
| *Vertical scramble,* upwards progression on multiple often oddly angled supports with no regular gait | 4.41 |
| *Extended-elbow vertical climb,* propulsion provided by hindlimbs (mainly through hip extension) with extended elbow forelimbs and some forelimb propulsion | 0.67 |
| *Bimanual pull-up,* body is lifted up by forelimbs using elbow flexion and humeral retraction | 1.72 |
| Vertical descent |  |
| *Rump-first symmetrical descent,* rump-first symmetrical descent, similar to flexed-elbow vertical climb | 9.27 |
| *Rump-first scramble descent,* as for ‘rump-first symmetrical descent’, but with no discernible gait often on multiple supports with odd orientations | 5.08 |
| *Rump-first forelimb only descent*,* rump-first descent in which only forelimbs are used to bear weight | 0.67 |
| *Rump-first cascade descent*,* as for ‘head-first cascade descent’, but rump-first | 0.82 |
| *Rump-first extended elbow descent*,* kinematic reverse of ‘extended-elbow vertical climb’ | 1.27 |
| *Fire pole slide,* rump-first sliding down vertical or subvertical support, usually larger than 20cm | 2.09 |
| *Head-first scramble descent,* head-first on multiple supports with odd orientations and no discernible gait | 0.15 |
| *Head-first cascade descent,* as for ‘head-first scramble descent’, except supports are smaller and radically angled | 0.07 |
| Torso-orthograde suspensory |  |
| *Brachiate,* classic hand over hand orthograde suspensory locomotion where forelimbs bear more than 50% of body mass with extreme trunk rotation, approaching 180° | 0.07 |
| *Forelimb swing,* similar to ‘brachiate’ but with little trunk rotation | 5.16 |
| *Flexed elbow forelimb swing,* as for ‘forelimb swing’ but with elbows flexed | 0.07 |
| *Orthograde transfer,* often begins with bimanual forelimb-suspension and may contain a lunge, where hands grasp small supports, after which a branch is pulled towards body with hand over hand or hand over foot motion. Weight is gradually transferred | 4.84 |
| *Orthograde clamber,* forelimb-suspensory where the hindlimbs assist and all four limbs are propulsors with the body weight borne by abducted forelimbs. Like brachiation but hindlimbs support in different orientations | 0.22 |
| *Arrested drop,* swinging from on top to underneath a support. | 1.79 |
| Torso-pronograde suspensory |  |
| *Inverted quadrupedal walk,* hands and feet used in combination with torso-pronograde and all limbs are in tension | 0.45 |
| *Inverted pronograde scramble,* as for ‘inverted quadrupedal walk’, but on irregular angled supports and no discernible gait | 0.15 |
| Forelimb-hindlimb swing |  |
| *Ipsilateral swing*,* swinging from ipsilateral fore- and hindlimb. Often as a single swing to join two other modes of locomotion | 0.15 |
| Bridge |  |
| *Cautious pronograde bridge,* torso-pronograde gap-closing movement where hands reach out to grasp support and cautiously pull body across | 0.60 |
| *Lunging bridge,* feet grasp a support, and a lunge closes the gap, with hands then grasping a distant support. | 0.45 |
| *Supinograde bridge,* as for ‘lunging bridge’, except suspensory | 1.27 |
| *Descending bridge,* an incomplete leap yielding hindlimb suspension that spans a gap followed by grasping support with forelimbs | 0.75 |
| Leap |  |
| *Pronograde leap,* torso-pronograde at take-off from either a posture or locomotion | 0.60 |
| *Pumping leap,* similar for ‘pronograde leap’ but with several forceful extensions of limbs to initiate branch sway which is used to add length to leap | 0.07 |
| *Vertical cling leap,* torso-orthograde clinging posture at take-off on a vertical support with push off mostly hindlimb powered | 0.30 |
| *Bipedal upward leap^1^,* differs from bipedal hop as hindlimbs push off simultaneously and there is a period of free flight, but progression is upwards and is only single bout rather than repetitive progression. Forelimbs can hold supports for stability but body weight is mostly borne on the hindlimbs | 0.75 |
| Drop |  |
| *Unimanual suspensory drop,* single forelimb suspension before drop, where body falls after releasing support | 1.49 |
| *Bimanual suspensory drop,* as for ‘unimanual suspensory drop’ but from both forelimbs | 0.90 |
| *Bipedal drop,* above branch bipedal balanced posture before drop | 0.45 |
| *Quadrupedal drop,* as for ‘bipedal drop’ but from quadrupedal posture | 0.07 |
| *Flexed elbow suspensory drop,* as for ‘suspensory drop’ but with forelimbs flexed | 0.07 |
| *Forelimb hindlimb suspensory drop,* drop from a forelimb and hindlimb suspension posture | 0.15 |
| Sway, gap-crossing movement used between supports by swaying a support where oscillations of increasing amplitudes using body weight and swayed until the animal can reach the other side of the gap. Also includes the oscillation of vertical branches and lianas | 0.22 |
| Ride, similar to sway but using supports to descend to lower levels in the trees or the ground | 1.64 |

*Submodes described by Thorpe & Crompton (2006)

^1^Newly identified locomotor behaviour for gorillas
